# Evolutionary Trends in RNA Base Selectivity Within the RNase A Superfamily

**DOI:** 10.3389/fphar.2019.01170

**Published:** 2019-10-09

**Authors:** Guillem Prats-Ejarque, Lu Lu, Vivian A. Salazar, Mohammed Moussaoui, Ester Boix

**Affiliations:** Department of Biochemistry and Molecular Biology, Faculty of Biosciences, Universitat Autònoma de Barcelona, Barcelona, Spain

**Keywords:** RNase, RNA, purine, catalysis, molecular dynamics, evolution, RNase A superfamily

## Abstract

There is a growing interest in the pharmaceutical industry to design novel tailored drugs for RNA targeting. The vertebrate-specific RNase A superfamily is nowadays one of the best characterized family of enzymes and comprises proteins involved in host defense with specific cytotoxic and immune-modulatory properties. We observe within the family a structural variability at the substrate-binding site associated to a diversification of biological properties. In this work, we have analyzed the enzyme specificity at the secondary base binding site. Towards this end, we have performed a kinetic characterization of the canonical RNase types together with a molecular dynamic simulation of selected representative family members. The RNases’ catalytic activity and binding interactions have been compared using UpA, UpG and UpI dinucleotides. Our results highlight an evolutionary trend from lower to higher order vertebrates towards an enhanced discrimination power of selectivity for adenine respect to guanine at the secondary base binding site (B2). Interestingly, the shift from guanine to adenine preference is achieved in all the studied family members by equivalent residues through distinct interaction modes. We can identify specific polar and charged side chains that selectively interact with donor or acceptor purine groups. Overall, we observe selective bidentate polar and electrostatic interactions: Asn to N1/N6 and N6/N7 adenine groups in mammals versus Glu/Asp and Arg to N1/N2, N1/O6 and O6/N7 guanine groups in non-mammals. In addition, kinetic and molecular dynamics comparative results on UpG versus UpI emphasize the main contribution of Glu/Asp interactions to N1/N2 group for guanine selectivity in lower order vertebrates. A close inspection at the B2 binding pocket also highlights the principal contribution of the protein ß6 and L4 loop regions. Significant differences in the orientation and extension of the L4 loop could explain how the same residues can participate in alternative binding modes. The analysis suggests that within the RNase A superfamily an evolution pressure has taken place at the B2 secondary binding site to provide novel substrate-recognition patterns. We are confident that a better knowledge of the enzymes’ nucleotide recognition pattern would contribute to identify their physiological substrate and eventually design applied therapies to modulate their biological functions.

## Introduction

The interest to solve a biological problem is frequently correlated to its inherent difficulty. When entering the RNA world we are faced with a wide diversity of secondary and tertiary structures. An even higher level of complexity is encountered when trying to identify the rules that guide the RNA binding protein recognition process. During the last decades, many efforts have been applied to unravel the structural determinants for protein RNA recognition (Draper, 1999; Allers and Shamoo, 2001; Draper, 2015; Terribilini et al., 2007). We are currently witnessing significant advances within the RNA field thanks to the novel RNA sequencing methodologies that have laid the path to an RNA-omics era. Nowadays, we have access to many protein-RNA binding predictors (Miao and Westhof, 2016) and the main basic rules that drive the protein–nucleotide interaction process have been identified (Luscombe, 2001; Denessiouk and Johnson, 2003; Morozova et al., 2006; Kondo and Westhof, 2011). The study of RNA cleaving enzymes poses additional complexity. Efficient RNases should first recognize a specific RNA target, and then provide a proper active site configuration to promote catalysis and ensure the proper cleavage of the substrate. A particular pharmacological interest relies on the design of tailored enzymes with specific RNA cleavage targets (Tamkovich et al., 2016). Recent work on RNases’ action within a cellular environment is helping to unravel their natural *in vivo* substrates (Honda et al., 2015; Lyons et al., 2017; Mesitov et al., 2017). A proper knowledge of the RNases’ active site architecture should lead to the design of specific inhibitors of their biological functions (Chatzileontiadou et al., 2015; Chatzileontiadou et al., 2018).

In this work, we have explored the nucleotide base preference within the vertebrate-specific RNase A superfamily. The bovine pancreatic enzyme RNase A was one of the earliest enzymes to be studied in the 20th century and is still one of the best characterized (Cuchillo et al., 2011). All the family members share a common three-dimensional fold, catalytic triad and mechanism of action on single-stranded RNA. During the last decades, the modular subsite arrangement of RNase A for the recognition of bases, ribose and phosphates has been characterized (Parés et al., 1991; Nogués et al., 1998). The enzyme cleaves the 3′5′ phosphodiester bonds with specificity for pyrimidines at the main anchoring site (B1) and preference for purines at the secondary site (B2) (Richards and Wyckoff, 1971; Raines, 1998). In a previous work, we analyzed the enzyme residues that were reported to participate in the specific binding of adenine (A) and guanine (G) bases at the B2 site among the RNase A superfamily members (Boix et al., 2013). A high evolutionary conservation was observed for B1, whereas a significant variability was visualized for the secondary base selectivity. Interestingly, the observed structural differences at the secondary base site correlate with their substrate specificity and catalytic efficiency (Tarragona-Fiol et al., 1993; Sorrentino, 1998; Boix et al., 2013). Likewise, the analysis of the protein conformational changes induced upon nucleotide binding by NMR and molecular dynamics highlighted an evolutionary trend in base interaction selectivity (Gagné and Doucet, 2013; Narayanan et al., 2017; Narayanan et al., 2018a). Conserved conformational rearrangements upon ligand binding within closely related members suggested a link between shared protein networks and their characteristic biological properties (Narayanan et al., 2018a). The RNase A superfamily includes a series of proteins with antimicrobial and immune-modulatory activities and is considered to have emerged with an ancestral host-defense role (Boix and Nogués, 2007; Rosenberg, 2008; Lu et al., 2018). Family members were classified according to their structural, enzymatic and biological properties into eight canonical types (Sorrentino and Libonati, 1997; Sorrentino, 2010). A better understanding of the structural determinants that govern the RNases’ substrate specificity can help us to explain the divergent functionalities within the family.

Here, we have committed ourselves to undertake a comprehensive comparative analysis of representative family members and explore the structural drift that has taken place through evolution to shape the substrate specificity of the secondary base binding site. First, we have performed a kinetic characterization of the first seven human canonical RNases using dinucleotides. Secondly, we have selected representative RNase A superfamily members from lower to higher order vertebrates and have performed molecular dynamics simulations of the protein-dinucleotide complexes.

## Materials and Methods

### Expression and Purification of the Recombinant Proteins

RNase A was purchased from Sigma Aldrich. The cDNA for RNase 1 was a gift from Prof. Maria Vilanova (University of Girona, Spain) and cDNA for RNase 5 was provided by Prof. Demetres Leonidas (University of Thessaly, Greece). RNase 4 synthetic gene was purchased from NZYtech (Lisboa, Portugal) and RNase 6 was obtained from DNA 2.0 (Menlo Park, CA, USA). RNase 2, RNase 3 and RNase 7 genes were obtained as previously described (Torrent et al., 2010). The recombinant proteins were expressed and purified as previously described (Boix, 2001; Prats-Ejarque et al., 2016). Briefly, the gene was cloned into the pET11c expression vector (Novagen), the protein was expressed in *Escherichia coli* BL21(DE3) cells (Novagen) and then purified from inclusion bodies. Finally, the protein was purified by cationic exchange FPLC on a Resource S column (GE Healthcare) and lyophilized. Protein purity was confirmed by SDS-PAGE and mass spectrometry.

### Spectrophotometric Kinetic Analysis

UpA, UpG and UpI (Biomers, Söflinger, Germany) were used as substrates, and the kinetic parameters were determined by a spectrophotometric method as described (Boix et al., 1999b). Assays were carried out in 50 mM sodium acetate, 1 mM EDTA, pH 5.5, at 25°C, using 1 cm path length cuvette. Substrate concentration was determined spectrophotometrically using the following extinction coefficients: ε_260_ = 24,600 M^−1^ cm^−1^ for UpA, ε_261_ = 20,600 M^−1^ cm^−1^ for UpG and ε_260_ = 16,400 M^−1^ cm^−1^ for UpI. The activity was measured by following the initial reaction velocities using the difference molar absorbance coefficients, in relation to cleaved phosphodiester bonds during the transphosphorylation reaction: Δε_286_ = 570 M^−1^ cm^−1^ for UpA, Δε_280_ = 480 M^−1^ cm^−1^ for UpG (Imazawa et al., 1968), Δε_280_ = 316 M^−1^ cm^−1^ for UpI (experimentally determined). Final enzyme concentrations were adjusted depending on the RNase activity for each assayed substrate in a range between 0.005 and 10 μM. The reactions were performed in triplicate with 100 μM of substrate and the activity was normalized at an enzyme/substrate ratio of 1:100.

### Molecular Dynamics Simulations

All the molecular dynamics (MD) simulations were performed using GROMACS 2016.2 (Abraham et al., 2015). The force field used was a modification of AMBER99SB (Best and Hummer, 2009). Charges of inosine were derived by R.E.D server (Vanquelef et al., 2011). The modifications of the force field to include inosine parametrization are detailed in the Supplemental Materials (**Figure S1**). All the complexes were centred in a dodecahedral cell with a minimum distance box-solute of 1.0 nm. The unit cell was filled with TIP3P (transferable intermolecular potential 3P) water (Jorgensen et al., 1983) in neutral pH conditions supplemented with 150 mM of NaCl.

Neighbor search was performed using a Verlet cut-off scheme (Páll and Hess, 2013) with a cut-off of 0.9 nm for both Van der Waals and coulombic interactions. For long range interactions, smooth particle mesh of Ewald (PME) (Darden et al., 1993; Essmann et al., 1995) was used with a fourth-order interpolation scheme and 0.1125 nm grid spacing for FFT. The bonds were constrained with the P-LINCS algorithm (Hess, 2008), with an integration time step of 2 fs.

The energy of the systems was minimized using the steepest descendant algorithm and equilibrated in two steps. First, an initial constant volume equilibration (NVT) of 1 ns was performed with a temperature of 300 K using a modified velocity rescaling thermostat (Bussi et al., 2007). Then, 1 ns of constant pressure equilibration (NPT) was run at 1 bar with a Berendsen barostat (Berendsen et al., 1984) at 300 K and the same thermostat. Finally, 100 ns production runs were performed under an NPT ensemble without applying restraints. Three independent simulations in periodic boundary conditions were conducted for each complex. Dinucleotides were generated by modifying the dCpA ligand of an RNase A–d(CpA) complex (Zegers et al., 1994), maintaining the same initial coordinates.

## Results

### Comparison of Canonical RNases’ Catalytic Activity on Dinucleotide Substrates: A Trend From Guanine to Adenine Selectivity At the B2 Secondary Base Site

**Figure 1:**
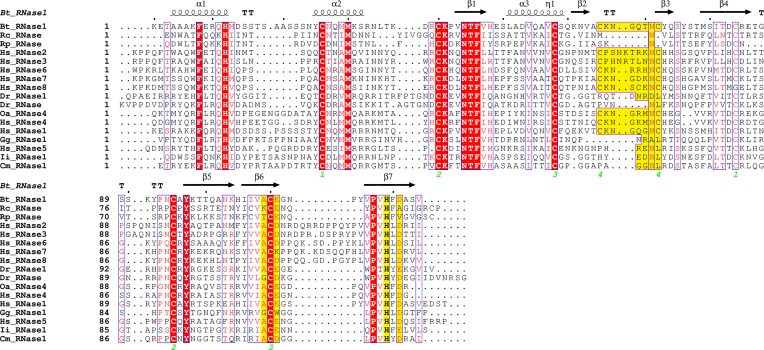
Sequence alignment of the eight canonical human RNases together with the RNase A superfamily members analyzed by molecular dynamics (sequences correspond to mature proteins, without the signal peptide). Protein regions identified to participate in B2 site are highlighted in yellow: L4, spanning from b2 to b3, end of ß6 (residues 109 and 111) and one of the two catalytic histidines together with a close by residue at ß7 (residues 119 and 121). TT indicates the presence of a β-turn. Dots label every 10 residues of the reference protein used (Bt-RNase 1, known as RNase A). The disulphide bonds are labeled with green numbers. Full species names are included in **Table S1**. Labels are as follows: red box with white character for strict identity; red character for similarity within a group; and character with blue frame for similarity across groups. The alignment was performed using *Clustal Omega* (Sievers and Higgins, 2018), and the picture was drawn using *ESPript* (Robert and Gouet, 2014).

In an effort to deepen into our knowledge of the evolutionary pressure that has guided the nucleotide base preference within the RNase A superfamily, we have compared the catalytic activity of the human canonical members on dinucleotides (**Figure 1**).

First, each canonical RNase was expressed using a prokaryote recombinant expression system. We successfully expressed and purified with high yield the first seven human canonical RNases using the T7 promotor and the pET expression system. Unfortunately, using the same prokaryote expression system we were unable to obtain a properly folded and catalytically active human RNase 8. In fact, inspection of the RNase 8 coding transcript by Rosenberg and co-workers revealed an unusual gene organization and protein disulphide pairing, suggesting a significant functional divergence from the canonical characteristic structure of the family (Chan et al., 2012). The authors do not discard the possibility that RNase 8 is not expressed as a standard secretory RNase. Therefore, we decided to perform our kinetic study using the first seven human canonical RNases. This is the first simultaneous comparison of the catalytic activity of all seven proteins within a single laboratory.

The catalytic activity of the RNases was assayed using dinucleotide substrates, where the first pyrimidine was kept invariable as a uridine and the secondary base was substituted by the natural standard purines and the modified base inosine. Together with the two natural purines incorporated in RNA during transcription, we have also selected inosine, a modified base frequently present in cellular RNA, as one of the main post-transcriptional modifications. Kinetic activity on UpA, UpG and UpI was measured by a spectrophotometric assay and the relative preference for the secondary base was estimated for each protein. Bovine pancreatic RNase A was taken as a reference control.

**Figure 2:**
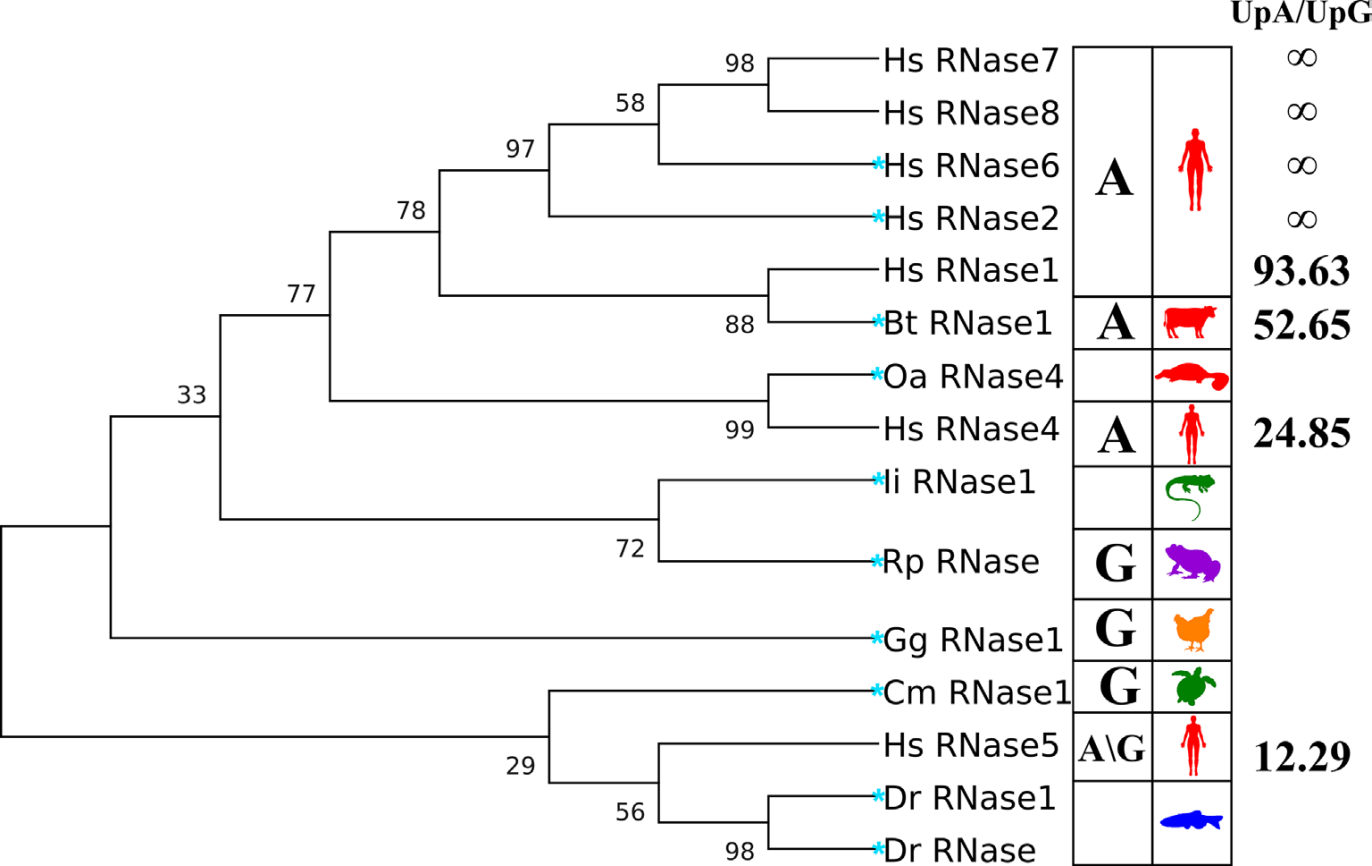
Phylogenetic tree of the RNases studied in the kinetic and molecular dynamics analysis. Right column indicates the UpA/UpG preference ratio according to kinetic results (see **Table 1**). Left column includes the A/G preference at B2 according to the literature. The evolutionary history tree was inferred by using the maximum likelihood method and JTT matrix-based model (Jones et al., 1992). The tree with the highest log likelihood (−3745.36) is shown. The percentage of trees in which the associated taxa clustered together is shown next to the branches. Initial tree(s) for the heuristic search were obtained automatically by applying Neighbour-Join and BioNJ algorithms to a matrix of pairwise distances estimated using a JTT model, and then selecting the topology with superior log likelihood value. The tree is scaled, with branch lengths measured as the number of substitutions per site. Evolutionary analyses were conducted in MEGA X (Kumar et al., 2018). A more complete phylogenetic tree of RNase A family members is included in **Figure S2**. Blue star highlights the selected representative RNases to perform MD simulation runs.

Interestingly, the respective catalytic activities of the seven human canonical RNases indicate a shift of the secondary base specificity, from a poor A/G discrimination to a pronounced preference for A (**Table 1**). In particular, the human RNase 5, which is the canonical member more closely related to ancestral RNases (Sorrentino, 2010), shows only a mild preference for adenine over guanine. In turn, the pancreatic-type RNase 1 shows a significant preference for adenine at B2 position. Last, the more evolved RNase subgroups (types 2/3 and 6/7) do not have any detectable activity using UpG as a substrate (**Table 1**, **Figure 2**).

**Table 1:** Kinetic activity of RNase A and the human RNases 1–7.

	V_0_ (µmol/min)			B2 ratio	
	UpA	UpG	UpI	A/G	G/I
Bt-RNase A	0.783 ± 0.033	1.49·10^−2^ ± 9.99·10^−4^	3.62·10^−2^ ± 2.22 ·10^−3^	52.65	0.411
Hs-RNase 1	0.108 ± 3.93·10^−3^	1.15·10^−3^ ± 6.09·10^−6^	7.87·10^−3^ ± 5.54 ·10^−4^	93.63	0.146
Hs-RNase 2	2.57·10^−3^ ± 1.32·10^−4^	n.d.	5.53·10^−4^ ± 1.56 ·10^−4^	∞	0
Hs-RNase 3	2.39·10^−3^ ± 4.96·10^−5^	n.d.	n.d.	∞	∞
Hs-RNase 4	5.92·10^−2^ ± 3.97·10^−3^	2.38·10^−3^ ± 2.86·10^−4^	6.86·10^−3^ ± 1.7 ·10^−4^	24.85	0.348
Hs-RNase 5	2.39·10^−4^ ± 3.74·10^−5^	1.94·10^−5^ ± 2.5·10^−6^	5.33·10^−5^ ± 8.19 ·10^−6^	12.29	0.365
Hs-RNase 6	6.74·10^−3^ ± 1.90·10^−4^	n.d.	n.d.	∞	∞
Hs-RNase 7	4.25·10^−5^ ± 6.21·10^−6^	n.d.	n.d.	∞	∞

The reactions were performed using 100 µM of substrate. Initial velocity (V_0_) of dinucleotide phosphodiester bond cleavage is indicated. The average of three replicates is shown. Standard error of the mean is shown. n.d.: not detected at the assayed conditions. Bt, Bos taurus; Hs, Homo sapiens.

On the other hand, when we analyze the kinetic characterization of other family members available in the literature, we can infer a shift at the substrate secondary base predilection, from lower to higher order vertebrates, from guanine to adenine (Boix et al., 2013). Basically, the characterized fish, amphibian and reptile RNases show a marked preference for G at B2 site (Hsu et al., 2003; Ardelt et al., 2008), while mammalian prefer A (Richards and Wyckoff, 1971; Zhao et al., 1998; Prats-Ejarque et al., 2016). We can group the family members, according to their relative activity on dinucleotide substrates, within three main subcategories by their base preference at the B2 site: G > A, G ∼ A and A > G (**Figure 2**). The results suggest that an evolutionary pressure has taken place to promote selectivity for the adenine base within the family’s more recently evolved members, coming from an ancestral precursor with a marked preference for guanine.

Last, we have studied the RNases’ activity on UpI dinucleotides. Inosine (I) was selected as an appropriate model to inspect the particular effect of the presence of a C=O group at the purine C6 atom and the influence of the NH_2_ group at the C2 position, in comparison to the other two purine base structures. Detectable activity for the inosine dinucleotide was mainly registered for the RNases 1, 2, 4 and 5 (**Table 1**). Overall, kinetic results indicate that no important differences are observed between the proteins’ enzymatic activity on UpG and UpI, although a slight preference for I over G is shown. Interestingly, the family members that have a closer relationship to lower order vertebrates (RNases 1, 4 and 5) present a significant activity against dinucleotides with inosine at the B2 position, but no detectable activity in the presence of a guanine. The results suggest that A/G discrimination within the RNase A superfamily relies partly in the recognition of N1/N2 group.

### B2 Base Selectivity Within the RNase A Superfamily by Molecular Dynamics

Following, to complement the enzymatic characterization of the canonical RNases, we performed a comparative analysis within the RNase A superfamily by molecular dynamics. To gain insight into the structural determinants of the protein recognition pattern at the B2 site, we have selected here representative members for each vertebrate family subgroup. Ten representative RNases were chosen and their binding mode to dinucleotides was predicted by MD simulations. **Figure 2** illustrates the selected proteins and their evolutionary relationships. When no solved 3D structure was available (RNase 4 of *Ornithorhynchus anatinus* and RNase 1 of *Iguana iguana*), a prediction model was generated using the *Modeller* software by the *ModWeb* server (Webb and Sali, 2016). From lower to higher order vertebrates, the following organisms were analyzed: *Danio rerio* (Dr), *Rana pipiens* (Rp), *Iguana iguana* (Ii), *Chelonia mydas* (Cm), *Gallus gallus* (Gg), *Ornithorhynchus anatinus* (Oa), *Bos taurus* (Bt) and *Homo sapiens* (Hs). Representative organisms were selected based on the available information on the evolutionary relationships and previous structure-functional characterization studies (Goo and Cho, 2013). We also indicate, when known, the canonical type of each selected RNase (Sorrentino and Libonati, 1997). Within the placental mammals, we have included two representative human members (RNases 2 and 6; UniProtKB P10153 and Q93091), which are expressed during infection and inflammation and are endowed with a high catalytic activity. We have also selected the bovine pancreatic RNase, or RNase A (UniProtKB P61823), which is the family reference member and one of the best characterized enzymes (Cuchillo et al., 2011). Bovine pancreatic RNase belongs to the RNase 1 type. On the other hand, for early mammals, we selected the platypus (*O. anatinus*), an egg-laying animal and precursor to higher order vertebrates, before divergence of placental RNases. Accordingly, the platypus RNase belongs to type 4 (UniProtKB F6ZXU1), and was previously identified as the predecessor of higher order mammalian RNase types (Goo and Cho, 2013). Following, representative members of avian, reptiles, amphibian and fishes were chosen, based on the availability of previously solved 3D structures. Chicken RNase 1 was taken (UniProtKB P27043) as the only member with a known 3D structure (Lomax et al., 2014). In turn, reptiles have been represented by turtle (UniProtKB P84844) and iguana (UniProtKB P80287) (Nitto et al., 2005). Next, we selected the northern leopard frog (*R. pipiens*) RNase (also named Onconase, UniProtKB P22069), which has been extensively characterized because of its antitumoral properties (Boix et al., 1996; Lee and Raines, 2003; Lee et al., 2008). Lastly, for fish representative sequences, we selected *D. rerio* RNases (Dr-RNase 1 and Dr-RNase; UniProtKB A5HAK0 and E7FH77), also named as zebrafish RNases 3 and 5 respectively. Both RNases were previously reported to display a high catalytic activity in comparison to other fish homologues (Cho and Zhang, 2007; Pizzo et al., 2011). In particular, the zebrafish 5 (Dr-RNase) was classified as one of the most ancestral family members, showing a high catalytic activity along with both antimicrobial and angiogenic properties (Pizzo et al., 2011). In all cases, previously reported 3D structures were taken as a reference, except for the platypus RNase, where a prediction model had to be generated.

To compare the RNases’ selectivity at the B2 site, the three dinucleotides, UpA, UpG and UpI, were selected (see **Figure S3** for atom nomenclature). Molecular dynamics were performed using GROMACS software as detailed in the methodology. Triplicates for each protein complex were carried out at 100 ns. The RMSD between the dinucleotide positioning during the simulation is shown in **Figure S4**. The following common criteria were established to confirm at the end of each modelling run that the nucleotide is positioned in a productive orientation, favorable for catalysis: phosphate location at the RNase catalytic triad and pyrimidine proximity to B1 site. Equivalent residues to RNase A (H12/K41/H119 at the catalytic triad and T45 at B1 site) were taken as a reference for each protein.

The dinucleotides’ RMSD fluctuations during each production run indicate a reduced substrate mobility, oscillating within a value range of 0.1–0.4 nm (**Figure S4**). The total hydrogen bond interactions per residue were calculated for each simulation and expressed as a fraction of occurrence. **Figure S5** illustrates the interacting residues with the purine moieties.

**Figure 3:**
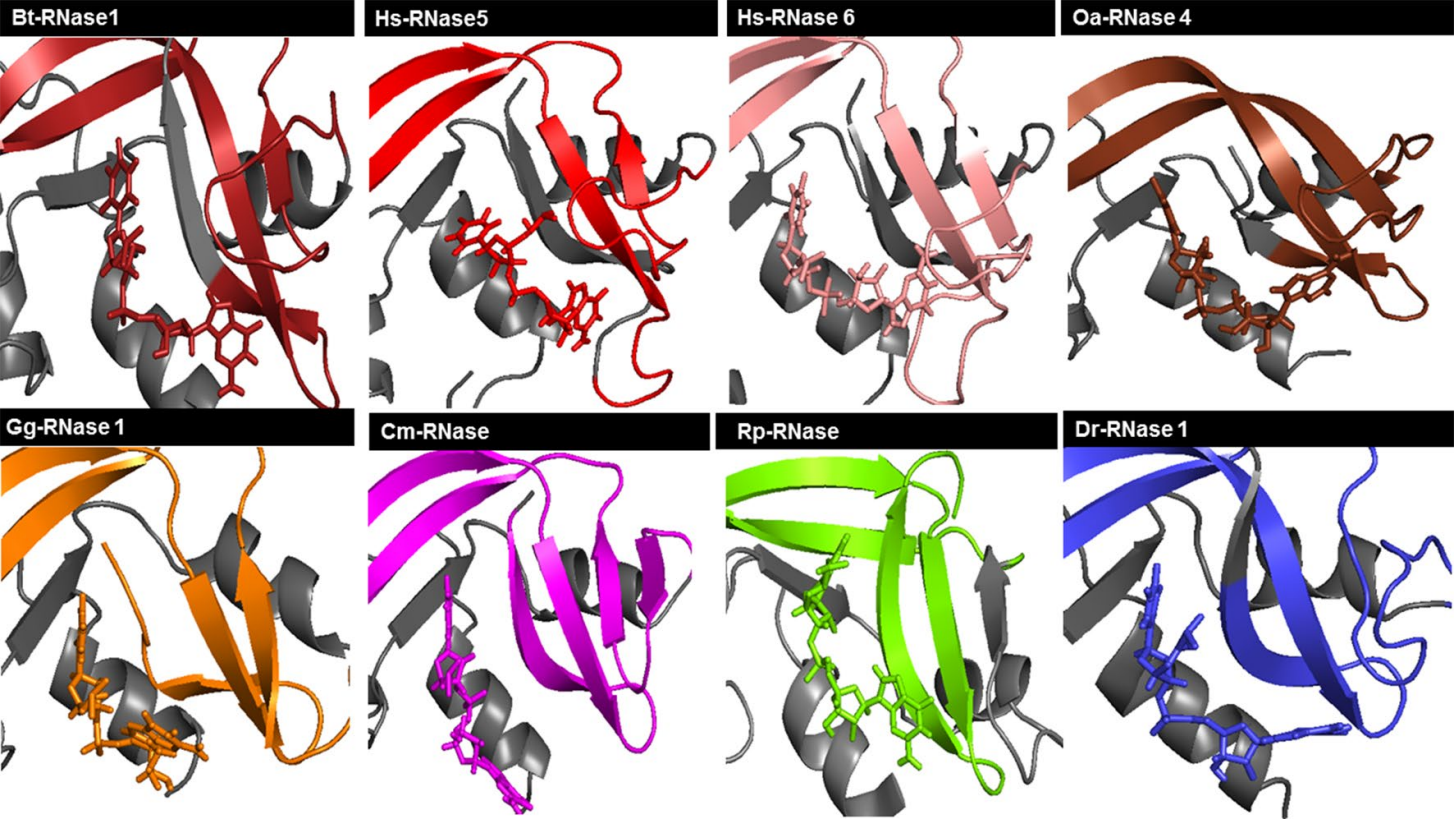
Schematic illustration of RNase–UpA complexes obtained at the end of the MD 100 ns simulation run. Colors of each vertebrate group are taken according to the family phylogenetic tree selection (see **Figure S2**). Mammal RNases are shown in shades of red, turtle RNase in magenta, chicken RNase in orange, frog RNase in green and fish RNase in blue. Some parts of the RNase are colored in gray to allow better visualization of the ligand and the interaction regions of the protein. The picture was generated using PyMOL 1.7.2 (Schrödinger, Inc).

**Figure 4:**
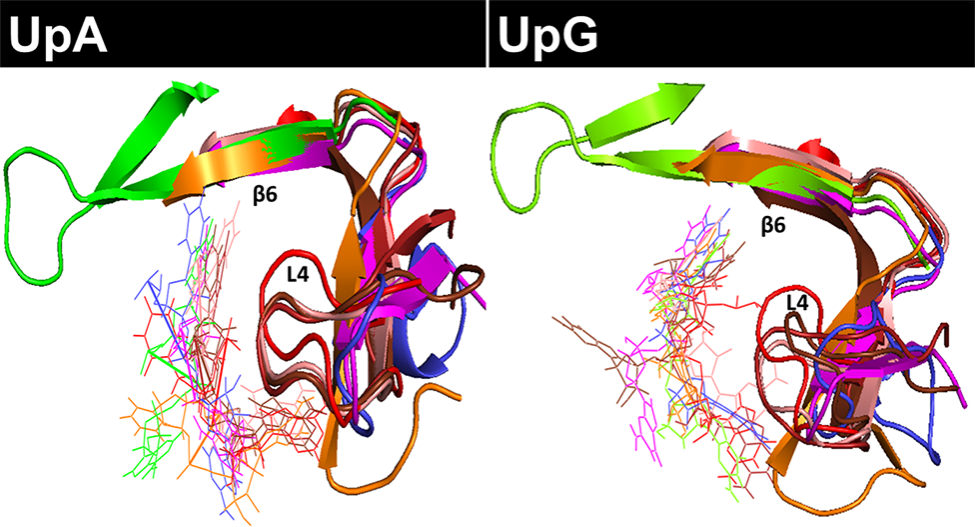
Overlapping of all RNase-UpA and RNase-UpG complexes obtained by molecular dynamics (see **Figures 3** and **S6**). The secondary base binding site (B2) is highlighted (loop L4 and strand ß6). As shown in **Figure 3**, the molecules are colored according to vertebrate groups as indicated in the family phylogenetic tree (see **Figure S2**). Mammal RNases are shown in shades of red, turtle RNase in magenta, chicken RNase in orange, frog RNase in green and fish RNase in blue.

Overall, we observe at the end of each simulation run a similar productive positioning of the dinucleotides at the active site cleft for most of the studied proteins (**Figures 3** and **S6**). However, comparison between all different RNase–nucleotide complexes and among triplicates highlights that most variability is located at the purine moiety (**Figure 4**). Likewise, time course analysis for each dynamic run shows significantly much higher mobility for the purine nucleoside in comparison to the pyrimidine main nucleoside and phosphate portions. We can confirm that the protein phosphate p1 and base B1 sites are mostly conserved among all the family members and provide stronger and more specific interactions.

**Figure 5:**
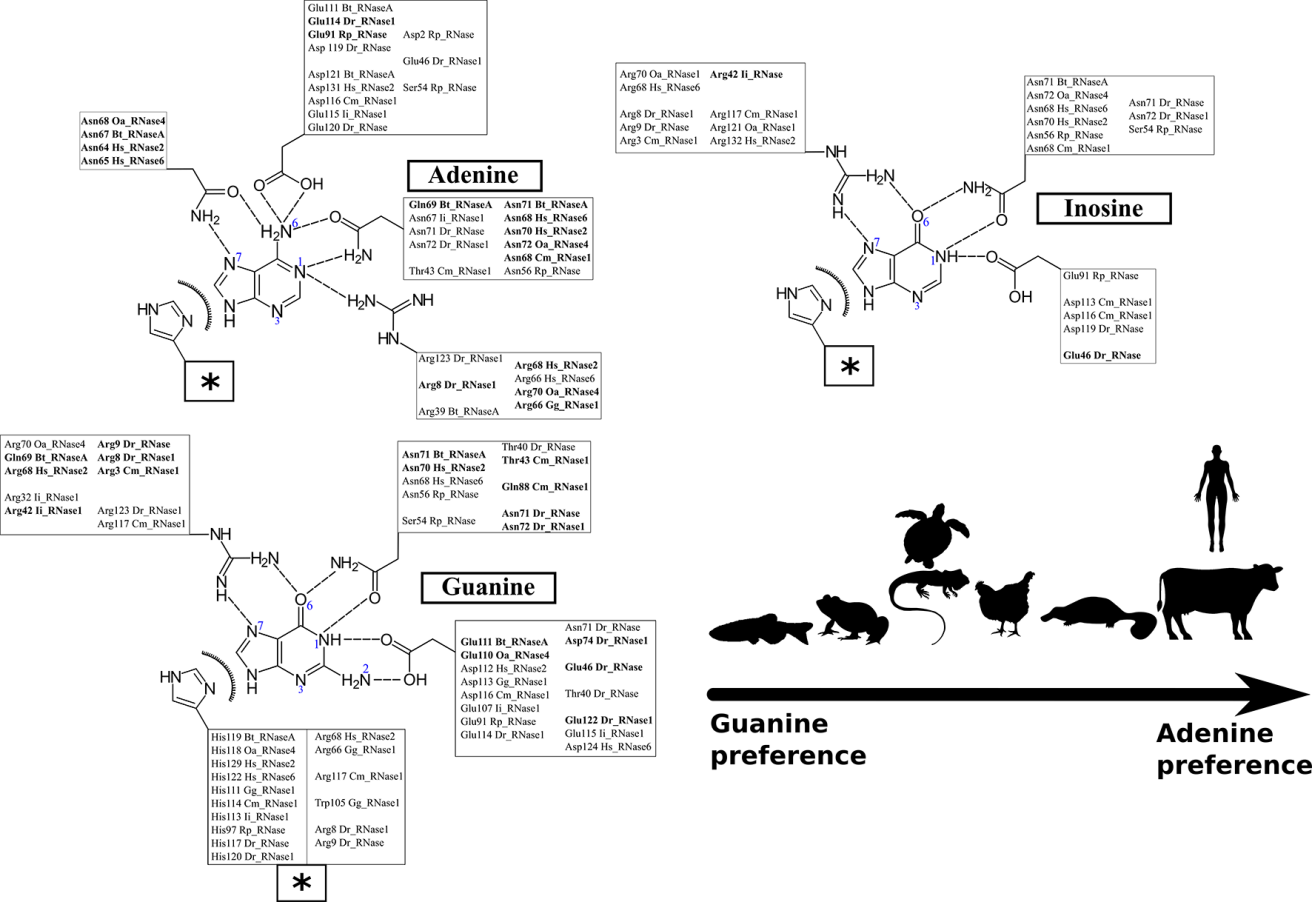
Schematic representation of the predominant interactions observed during the 100 ns of each simulation run. Only the interactions present during more than 30% of the total dynamic runs were considered. The interactions observed in more than 50% of the total dynamic runs are highlighted in bold. Protein residues are listed according to RNase A numbering reference (see **Table S2** for structural residues overlapping between the analyzed homologues). Polar and electrostatic interactions are detailed for each location. The box labeled with a star includes the shared common van der Waals interactions for the three base types.

Following, we have analyzed the specific binding interactions at the B2 purine portion. Specific binding residues at the B2 site were identified. In the majority of complexes, the purine base is fixed by the L4 loop and ß6 strand structures (**Figure 4**). Contribution of each interacting residue was monitored as a function of time. Each run was subdivided into initial, central and late periods. Although some mobility of the substrate positioning is observed during the 100 ns MD production runs (**Figure S4**), overall no major significant differences are identified as a function of time. The most representative interacting residues and atom types involved in each modelled complex are summarized in **Figure 5**.

**Figure 5** illustrates the main residues that contribute to B2 base recognition. The figure indicates the main residues that were found involved in interactions with the purine ligand for at least one third of the total 100 ns molecular dynamics run. We observe the contribution of polar and charged residues that act as acceptors/donors to purine representative groups. We can identify the protein residues that can provide a bidentate anchoring with the purine base and selectively interact with unique base groups. In particular, we find specific discriminators for adenine (N1/N6 and N6/N7 groups) versus guanine (N1/O6 and N1/N2 groups). Likewise, discrimination between guanine and inosine binding was identified by looking for the residues with specific interactions at the base N1/N2 group, unique to guanine.

Each studied family member was analyzed taking bovine pancreatic RNase A sequence numbering as a reference (see **Table S2** and **Figure 1**) (Raines, 1998; Boix et al., 2013). The adenine base is fixed in bovine RNase by residues Asn67, Gln69, Asn71, Glu111 and His119 (see **Figure 5**). The reliability of the dynamic simulation was first evaluated by comparing the obtained results for RNase A using UpA with the previous structural work by X-ray crystallography on RNase A–dinucleotide complexes (Boix et al., 2013). In particular the adenine binding residues identified by molecular dynamics were compared with the RNase A–d(CpA) complex (Zegers et al., 1994), where the same residues for adenine binding had been identified (Asn67, Gln69, Asn71, Glu111 and His119). Specific bidentate interactions for adenine are provided by Asn71/Gln69 at N1/N6, Asn67 at N6/N7 and Glu111 at N6. In particular, our molecular dynamics results corroborate the key contribution of all Asn71 counterparts in mammalian members for adenine specificity. On the other hand, we observe the flexibility of residues such as Glu111, which can offer a bidentate anchoring at either the NH_2_ group at C6 position in adenine or at N2/N1 groups in guanine (**Figure 5**).

In addition, we observe the contribution of the His119 catalytic residue by π-π interactions with the purine 5-membered ring in all the predicted complexes for any of the three assayed dinucleotides (**Figure 5**). Previous structural studies have revealed that the catalytic His119 in the free protein can adopt two conformations (A and B), where only one rotamer (A) is compatible with catalysis and purine interaction (Berisio et al., 1999; Merlino et al., 2002). Favored stacking interactions of the His imidazole with the purine ring are suggested to participate in nucleotide discrimination (Gagné and Doucet, 2013). In our molecular dynamics study we cannot find any significant differences between the complexes obtained with any of the three dinucleotide types. On the other hand, significant differences are observed for some particular RNases, where the purine ring is also establishing cation-π interactions with other residues, in particular arginine (such as Arg68 in Hs-RNase 2, Arg66 in Gg-RNase, Arg117 in Cm-RNase and Arg8 in Dr-RNase 1 (see **Figure 5**). Overall, we observe that most differences among the studied family members are located at the L4 Loop. The loop mobility is restricted by a disulphide bridge (Cys65–Cys72 pair in RNase A), that is conserved in most mammalian RNases (except in RNase type5/angiogenin-like), but absent in all the non-mammalian vertebrate groups (see **Figure 1**).

We can conclude from the analysis of predicted protein–dinucleotide complexes that the main key residues for purine interactions (Asn71 and Glu111) are mostly conserved among all the studied family members, although distinct binding modes are identified depending on the nature of the purine base. Asn71 in RNase A, and equivalent residues both in human and platypus proteins, specifically bind by a bidentate interaction at the N1/N6 of the adenine ring. Likewise, the Asn side chain can establish equivalent interactions for guanine or inosine binding, by shifting their NH and C^=^O amide groups and thereby interacting with the respective N1/O6 groups. However, these interactions are not so often observed for guanine/inosine interaction and frequently only the Asn binding to the O6 group is identified.

When we inspect the non-mammalian vertebrate members, we find a similar scenario: an Asn residue (Asn71 RNase A counterpart) can also interact with both N1/N6 groups for A and N1/O6 in G/I in turtle, frog and fish proteins. Significant differences are found in chicken RNase, where an Arg is located at the same position. On its turn, the nearby residue Gln69 would contribute to provide a specificity for adenine. A Gln at this position is only present in the pancreatic RNase 1 type. Substitutions of Gln by an Argin Hs-RNases 2 and 6 and platypus RNase favor the bidentate interaction with G/I at the N7/O6 group. The equivalent counterpart in fish is an Asn (), which shows a preference for guanine/inosine binding. No equivalent residues are found in any other lower order vertebrates, due to a deletion in the loop L4 region from residues 65 to 71 (see **Figures 1** and **S7**–**S11**). In addition, we find another Asn residue in mammalian RNases that is also favoring the adenine versus guanine binding: Asn67 (**Figure 5**). In this case, the Asn is providing a bidentate interaction to N6/N7 adenine groups. The presence of an additional Asn is also found in zebrafish RNase 5 (Dr-RNase) but is missing in all the other studied lower order vertebrates. Interestingly, the shorter L4 loop version in the fish protein still permits the appropriate Asn positioning.

The molecular dynamics results also highlight two other protein regions, which are also participating in the purine binding: residues 109–111 (ß5) and 119–121 (ß6). In particular, we observe the main contribution of Glu111 in Bt-RNase A and the respective Glu/Asp counterparts in the other studied family members (**Figure 5** and **Table S2**). Both the Glu/Asp bidentate anionic side chains are observed to bind at both the NH_2_/N6 adenine and the N2/N1 guanine specific groups. However, Glu substitution by an Asp residue (found in Hs-RNases 2 and 6) prevents, or reduces drastically, the base interactions. Similar interactions at the adenine N6 NH_2_ group and the guanine N2/N1 group are established by Asp121 at Bt-RNase A and their counterparts in mammals and chicken RNases. Although a Glu/Asp is present in all the studied proteins, frog RNases show significant differences. Interestingly, the zebrafish 3 counterpart (Glu122) interacts with guanine base but is not involved in adenine binding. Finally, another substitution that is observed to favor guanine binding in non-mammalian RNases is Ala122 to Arg. The Arg counterpart residues in fish and turtle RNases can interact by bidentate interactions with the O6/N7 group of the guanine/inosine bases (**Figure 5**).

Overall, although key residues for purine binding are mostly conserved in all the studied members, such as Asn71, His119 and Glu111, our molecular dynamics analysis indicates that distinct binding modes could promote a shift from G to A at the B2 site.

### An Evolutionary Trend Shaping the B2 Selectivity Within the RNase A Superfamily Lineage

To validate the significance of the residues identified by MD to participate in purine recognition, we have supplemented our study with the comparative analysis of other family member close homologues. Accordingly, each representative member analyzed by MD simulations has been compared within its own vertebrate subgroup. By close inspection of sequence alignments, we have identified the counterpart to the key residues for binding of a purine at B2 location. **Figures S7**–**S11** include the respective sequence alignments within each vertebrate subgroup. The relationships between all the aligned sequences of family homologues are illustrated in the phylogenetic tree included in **Figure S2**.

First, we have analyzed the fish RNase sequences, taking as a reference *D. rerio* RNase 1 (Pizzo et al., 2011), also named zebrafish RNase 3 (ZF3). Acharya and co-workers solved the crystal structure of this RNase together with a polymorphism variant (Kazakou et al., 2008). The chosen protein structure corresponds to the variant identified as ZF3e. Overall, the researchers identified five protein variants, with substitutions at six sequence locations. Among them, we observe that one of the residues involved in the purine binding (Arg123) is only present in the ZF3e polymorphism and is substituted by a Lys in the other variant. On the other hand, comparison with the other fish RNase sequences (**Figure S7**) highlights the presence of one or two conserved Asn residues at L4 loop region. The loop is present in fishes in a short-reduced version in comparison to the extended version present in more evolved mammal RNase types: 2/3–6/7/8 (**Figure 1**). However, the Asn residue at position 72/74 (corresponding to positions 67 and 71 in RNase A) can also participate in the adenine interaction but would preferably interact with the N1-O6 group of a guanine. Noteworthy, several fish RNase sequences display an Asp at 74 position, which according to molecular dynamics results is a suitable binder for guanine. Two other anionic residues at the protein C-terminus are key for the studied RNase complexes; that is Glu114 and Glu122 (corresponding to Glu111 and Asp121 in RNase A counterparts). While most fish RNases show a Glu at 114 position, we also find in some cases the presence of an Asp. This is the case of zebrafish 5 (Pizzo et al., 2011), which was reported to have a relative much higher catalytic activity than the other characterized fish RNases (Pizzo et al., 2011). Likewise, residue Glu122 is either conserved or substituted by an Asp residue. Finally, molecular dynamics reveal the presence of an Arg residue at the zebrafish proteins’ N-terminus that shows favored interactions to guanine and inosine. The Arg is only present in about 50% of the analyzed fish sequences.

Following, we inspected the residues potentially involved in purine binding in amphibians. In this vertebrate group we also observed a short version of the L4 loop. However, in comparison to fish RNases, the analyzed amphibian members show a less optimal loop conformation. The loop is orientated to the opposite direction respect to RNase A, and lacks one of the key Asn found in mammalian RNases. In particular, in northern leopard frog *R. pipiens* RNase (Onconase) we can identify Asn56 (counterpart of Asn71 in Bt-RNase A) but no other equivalent residues in the region (**Figure 1**). Comparative structural alignment only reveals the presence of a conserved Glu residue at position 91 (Glu111 counterpart in RNase A). Molecular dynamics results on Onconase interaction with dinucleotides have been compared with the previous reported solved crystal structure in complex with a tetranucleotide (Lee et al., 2008). Raines and co-workers studied in detail the enzyme binding to the d(AUGA) substrate analogue and observed that while an equivalent binding pocket is conserved for the pyrimidine base at B1, significant differences are found for the B2 site. In particular, specific bidentate interactions of Glu91 with the guanine base were identified at B2 position. Moreover, the authors confirmed by site-directed mutagenesis that this residue was responsible for the frog RNase preference of guanine over adenine. In addition, the authors also highlighted the importance of the nature of the nearby residue located at position 89 (Onconase counterpart of Ala109 in RNase A). Ala109 is conserved in all mammalian and most reptile sequences but shows a significant variability in fishes and amphibians. Interestingly, ZF3 presents an Ala at this position, as observed in mammals, whereas other fish RNases have a polar or cationic residue (Thr/Lys or Arg), as observed in frog RNases. Substitution of Thr89 in Onconase by an Asn residue reduced the enzyme’s G > A preference. The authors suggested that long-range electrostatic interactions were key for the enzyme turnover activity on cellular RNA substrate in physiological conditions (Lee et al., 2008). The hypothesis was further backed up by recent NMR and molecular dynamics studies by Doucet and collaborators, that emphasized the key role of network interactions connecting distant protein residues (Narayanan et al., 2018a). Interestingly, site-directed mutagenesis in Onconase revealed also the contribution of the N-terminus in the B2 base discrimination (Lee et al., 2008). In particular, insertion of an Arg at position 5 is significantly enhancing the frog RNase catalytic activity. Likewise, in our molecular dynamics analysis we observe equivalent Arg residues at the protein N-terminus of turtle and fish RNases that contribute to purine binding (**Figure 5** and **Table S2**).

Molecular dynamics results of Onconase were also compared with the structural information reported for bullfrog (*R. catesbeiana*) RNase purified from oocytes (RC-RNase), the most catalytically active frog RNase (Chang et al., 1998; Lee and Raines, 2003). A structural study by NMR of bullfrog oocyte RNase analyzed the enzyme interaction with tetranucleotides (Hsu et al., 2015). The authors reported a much higher catalytic activity for oocyte RC-RNase in comparison to RC-RNase 2 and RC-RNase 4. The contribution of the L4 loop to guanine binding was also highlighted, although distinct conformations are observed among the bullfrog RNases that could account for the higher catalytic activity displayed by the oocyte RC-RNase. When we overlap the reported NMR structures with our modelled structures in complex with dinucleotides, we also observe that the oocyte RC-RNase is the only one that has an Asn residue at an equivalent position to Asn71 in RNase A, that can establish interactions with the N1-O6 group of the guanine. Therefore, the higher catalytic efficiency of bullfrog oocyte RNase respect to Onconase could be mostly attributed to residue Asn57, which is substituted by a Ser in the latter. When we compare the sequence identities of the distinct frog RNases, we observe a high variability at the loop L4, where Asn residues are mostly substituted by either a Ser or an Asp (**Figure S8**). Besides, presence of Pro and short amino acid insertions in other amphibian RNases might also modify significantly the interaction at this site. Interestingly, whereas most *R. catesbeiana* RNases show a particular four amino acid insertion, we found several *Xenopus* species that display an alternative loop version, with a slightly extended insert (**Figure S8**).

In turn, reptiles present a short version of the L4 loop (see **Figure S9**), with a similar length to the one observed in fishes, although encompassing a higher sequence divergence at the region. In particular, most species include only one Asn within the region. In our molecular dynamics study of iguana and turtle RNase-dinucleotide complexes we can identify one Asn (Asn68 in turtle RNase and Asn67 in iguana) equivalent to the Asn71 counterpart in RNase A (**Table S2**). Close inspection of sequence alignment identifies few reptile species with two Asn residues at 67/71 positions (such as the species of the *Micrurus* or *Boiga* genera), whereas other species show an Asn to Asp substitution at position 71. However, we observe an overall higher variability at L4 loop, which incorporates non-conserved substitutions (**Figure S8**). We have also analyzed within reptiles the other residues that were identified in turtle or iguana to potentially participate in binding at the B2 site (**Figure 5**). Rosenberg and co-workers characterized the RNase from iguana, which is mostly expressed in the pancreas and displays a significantly high catalytic activity (Nitto et al., 2005). In the present work, productive binding conformations obtained by molecular dynamics of turtle RNase with dinucleotides highlight the contribution at the protein C-terminus of Asp116 and Arg117 (Asp116, counterpart of Asp121 in RNase A, is only present in few reptile sequences). In turn, the presence of an Arg at position 117, not shared by all the family homologues, is rare.

Avian RNases present the shortest L4 loop version that incorporates the most significant deviation from the L4 loop consensus sequence (**Figure S10**). Most L4 sequences do not include any Asn residue. In our modelled complex of chicken RNase, Asn65 is equivalent to Asn67 in RNase A. However, we did not observe any direct participation of Asn65 in purine binding. In turn, the neighboring residue Arg66 is significantly participating in B2 binding and was observed to bind to any of the three purine bases. Arg66 position can be equated to Bt-RNase A Asn71 counterpart, although the loop conformation is very divergent at this region (**Table S2**). Arg66 is only found in few bird sequences but is located close to Arg66/70 in some other mammalian RNases (Hs-RNase 2, Hs-RNase 6 and Oa-RNase in our study; **Figure 5** and **Table S2**). Most strikingly, there is no equivalent Glu/Asp counterpart to Bt-RNase A Glu111. On the contrary, a Trp is present at that location in the studied chicken RNase (**Figure 1**). Trp is conserved in some chicken and snake (*Boiga*) RNase sequences (**Figures S9**, **S10**). In other avian sequences we find another bulky hydrophobic residue, followed by Asp, which might substitute the Glu111 function. Interestingly, in our molecular dynamics study we find the contribution of stacking interaction of Trp105 with the purine base. On the other hand, the presence of a residue equivalent to Asp121 is only observed in some of the sequences, whereas others show a substitution by an Ala. In any case, a higher proportion of non-productive dinucleotide binding is obtained by molecular dynamics (>75% of all run assays) in relation to the other studied members (<30% in fish, frog, turtle or platypus), which might be attributed to the chicken RNase’s different conformations of the L4 loop and the presence of Arg66 and Trp105, that tend to establish stacking interactions with the purine base. Noteworthy, Rosenberg and co-workers performed a comparative study of available sequences for chicken RNases and concluded that the evolution within this group of proteins might not respond to functional constraints directly related to the enzyme catalytic activity (Nitto et al., 2006). Comparison of two chicken leukocyte RNases identified key regions for either antimicrobial or angiogenic activity. By construction of hybrid proteins, they concluded that following a duplication event, a selective evolutionary pressure unrelated to the protein enzymatic activity had taken place. In our study, we have selected the only available 3D structure of a chicken RNase. Unfortunately, this RNase corresponds to the angiogenin-type RNase instead of the other characterized chicken RNase (leukocyte RNase-A2 or RSFR-RNase), which displays a much higher catalytic activity (Nitto et al., 2006).

In contrast to lower order vertebrates, we observe that all mammalian RNases, except the RNase 5 type, share an extended L4 loop fixed by a disulphide bridge (Cys65 and Cys72 in RNase A). In contrast, all the non-mammalian vertebrate RNases have either a single Cys or none at this location (**Figure 1**). Mammalian RNases’ extended L4 loop includes in all cases the RNase A Asn71 counterparts and, in the majority of cases, the RNase A Asn67 residue. On the other hand, more variability is observed at 69 position, where either a Gln, Ser or Arg is found (**Figure S11**). On its side, Glu in position 111 is mostly conserved but can also be substituted by an Asp or even a Lys residue. Our MD results indicate that the presence of the shorter Asp residue is associated in Hs-RNase 2 and Hs-RNase 6 with scarce or null interactions with the purine (**Figure 5**). Structural crystallographic data on Hs-RNases 2 and 3 complexes (Mohan et al., 2002) also highlighted the different interaction mode of Asp at this position. Hs-RNase 6 structural studies also indicated that the Glu to Asp substitution might significantly modify substrate specificity (Prats-Ejarque et al., 2016). On the other hand, it is also interesting to note the presence of an Arg at position 122 (Arg132 in RNase 2), which is shared with some fish and other lower order vertebrates (**Table S2**) and might provide a significantly differentiated specificity. Overall, we can conclude that counterpart residues to Asn71 and Glu111 in Bt-RNase A, shared by all the mammalian RNases, were already present in most ancestral RNases; but the observed purine selective specificity is modulated in each family member by complementary interactions of environment residues.

## Discussion

The RNase A superfamily is currently a reference model for evolutionary and enzymology studies. Although a wealth of information is available on ruminant evolution and the pancreatic-type RNases (Beintema and Kleineidam, 1998; Goo and Cho, 2013; Xu et al., 2013; Lomax et al., 2017), a comprehensive full understanding of the whole family is still missing. Following a pattern characteristic of host-defense proteins, the RNase A family has undergone frequent duplication and gene sorting events (Rosenberg et al., 1995; Zhang et al., 2000; Zhang et al., 2002; Liu et al., 2015). Many studies have tried to unveil the structural determinants for the distinct RNases’ biological activities (Lu et al., 2018); however, we find much less information on the evolutionary trends that shaped the family’s enzymatic diversity. Nonetheless, the understanding of the evolutionary processes that determined the enzymes’ substrate selectivity is key to unravel their physiological roles. Distinct nucleotide specificities should respond to an adaptation to their respective biological functions (Narayanan et al., 2018b). Undoubtedly, mastering the structural basis for protein nucleotide recognition is essential to assist the design of novel anti-infective and immunomodulatory drugs.

Here, we have compared for the first time the catalytic activity of the human canonical RNases. The analysis of all the recombinant proteins, obtained by the same expression system and using the same kinetic characterization methodology, ensures a reliable comparative evaluation of their respective efficiencies. To unravel the enzyme specificity for the binding of the purine secondary base, we have tested the respective catalytic activity of the distinct canonical RNases using UpA, UpG and UpI dinucleotides as a substrate. Interestingly, when we compare the A/G ratio at B2 site for the studied seven canonical RNases (**Figure 2**), we observe a pronounced evolutionary tendency from guanine to adenine preference. Previous evolutionary studies identified within the RNase A superfamily the phylogenetic relationship between the eight canonical subtypes (Cho and Zhang, 2007). By comparative analysis of the family coding sequences we can order the different RNase types from ancestral to modern as follows: 5, 4, 1 and 6/7/8–2/3 group (Zhang, 2007; Sorrentino, 2010) (see the family phylogenetic tree in **Figure S2**). Kinetic results of human canonical RNases follow the same ordering when considering the UpA/UpG ratio (**Table 1**). The present result is in agreement with previously reported kinetic data, where lower order vertebrates, such as amphibians and reptiles, show a preference for G (Liao, 1992; Irie et al., 1998), while mammalian RNases have a clear preference for A (Boix et al., 2013). In addition, the recent kinetic characterization of human RNase 6 corroborated the previously reported preference for adenine at B2 for human RNases 2 and 3 (Boix et al., 1999a; Sikriwal et al., 2007; Prats-Ejarque et al., 2016). On its turn, human RNase type 5 shows a much less pronounced preference for adenine over guanine (Acharya et al., 1994; Shapiro, 1998). Early kinetic characterization of human RNase 5 (angiogenin) already reported its poor discriminating ability on the purine located at B2 position (Shapiro et al., 1986; Harper and Vallee, 1989). Vallee and co-workers engineered an RNase 5 hybrid protein by replacing the L4 loop residues 60-70 with the RNase A counterparts, successfully enhancing the enzyme catalytic activity on UpA dinucleotides (Harper and Vallee, 1989). Further work by site-directed mutagenesis in angiogenin suggested that an Asn at Gln69 position in RNase A would provide most of the purine selective binding. On the contrary, replacement of the Glu111 RNase A counterpart (Glu108 in RNase 5) did not significantly alter the enzyme B2 selectivity (Curran et al., 1993). Indeed, structural and molecular dynamics simulation studies indicate that Glu111 in RNase A could contribute to either adenine or guanine binding by alternative modes, by direct or water-mediated interactions. The nature of the nearby residue (109 in RNase A) could determine the potential participation of the corresponding Glu residue (Glu111 in RNase A) in the purine binding. The hypothesis was elegantly confirmed by site-directed mutagenesis studies in Onconase (Lee et al., 2008). Raines and co-workers demonstrated that the B2 site specificity could be shifted from guanine to adenine preference by impeding the long distance network interactions that Glu establishes for the purine recognition (Lee et al., 2008). Likewise, substitution of Glu111 by the shorter Asp side chain in human RNases 2 and 6 could enhance the adenine versus guanine discriminating power in relation to ancestral RNases, such as RNase-type 4 and 5, as observed in our kinetic comparison studies (**Table 1**, **Figure 2**).

In an effort to unravel the structural determinants underlying the observed differentiated kinetic behaviors, we have carried out a molecular dynamics analysis of RNase-dinucleotide complexes. Representative family members were chosen from lower order vertebrates to placental mammals. Overall, molecular dynamics corroborate the observed shift from guanine to adenine preference by kinetic analysis (**Table 1**, **Figure 2**). Notwithstanding, results also highlight that conservative sequence identities are frequently not accompanied by equivalent substrate binding. A similar conclusion was reached by NMR analysis of RNases’ nucleotide binding (Narayanan et al., 2018a). Therefore, no straightforward conclusions can be directly inferred from the identification of individual interactions to nucleotides by mere structural overlapping analysis. On the other hand, although molecular dynamics considers the protein–ligand complex as an entity in motion and provides the equivalent freedom and flexibility that could be found in experimental conditions, the methodology has also its own limitations when trying to simulate the enzyme behavior. Fortunately, Bt-RNase A, the family reference member, has been one of the best enzymes ever characterized (Raines, 1998; Cuchillo et al., 2011). RNase A was classified by earlier studies as an almost “perfect” enzyme, where the transphosphorylation state is not limited by the transition state (Albery and Knowles, 1976). Raines and coworkers analyzed the behaviour of RNase A on UpA substrate by experimental kinetics and concluded that the cleavage efficiency is mostly limited by the substrate desolvation (Thompson et al., 1995). Early crystallographic and NMR studies of RNase A in complex with mono-, di- and tetranucleotides identified the main residues that conformed the RNase A substrate binding subsites (Fontecilla-Camps et al., 1994; Nogués et al., 1998; Hsu et al., 2015).

Notwithstanding, despite the RNase protein small size and structure stability, that facilitated the pioneer biochemistry works during the first half of the 20th century, the polymeric nature and structural complexity of the substrate is still challenging the enzymologists. In this context, it is important to analyze the protein family members as a whole dynamic entity. The protein has a kidney-shaped structure conformed by two domains that delimitate the catalytic active site groove. The open and closed conformation of enzymes were compared in the presence of nucleotide ligands (Watt et al., 2011; Gagné and Doucet, 2013). Key residues involved in the RNase protein motion would have co-evolved to shape the enzyme catalytic efficiencies, as described for other enzyme families (Maguid et al., 2006; Ramanathan and Agarwal, 2011; Narayanan et al., 2018a). Within the RNase A superfamily we observed the conservation of key domains involved the protein motion (Merlino et al., 2003; Gagné and Doucet, 2013). Notwithstanding, comparative studies from lower to higher order family members infer an inverse relationship between the protein’s structural rigidity and its catalytic efficiency (Merlino et al., 2005; Holloway et al., 2011).

Although our molecular dynamics runs using dinucleotides are overall in agreement with the reported crystal complex structures (Fontecilla-Camps et al., 1994; Zegers et al., 1994; Leonidas et al., 2001; Mohan et al., 2002; Lee et al., 2008), we do observe some significant differences. This might be due to the allowed protein flexibility during the molecular dynamics simulations, a fact that could enable a better accommodation of the nucleotide substrates. Besides, MD studies permitted us to work with the natural enzyme substrates, rather than the analogues, commonly used in crystallographic studies. On the other hand, NMR titration studies using mononucleotides could only mimic the enzyme interactions that were to take place with the enzyme reaction product (Narayanan et al., 2018a). Interestingly, when we analyze the results of our molecular simulation, we can observe significant differences among the residues that participate in the distinct periods of the reaction. Mostly, interactions with the purine base are frequently lost at the end of the production run. Interestingly, in our modelling studies we observe how the substitution of Glu111 by an Asp residue in human RNases 2 and 6 is only participating in the purine binding at the initial step of the reaction. In contrast, the positioning of the pyrimidine base, located at the main B1 site, and the phosphate are mostly retained during all the simulation run, as reported in previous molecular dynamics using RNase A or angiogenin (Madhusudhan and Vishveshwara, 2001). Indeed, Raines and co-workers’ kinetic studies indicated that the RNase A catalytic mechanism relies mostly on the substrate association step (delCardayre and Raines, 1994). A high catalytic efficiency would mostly be associated to the enzyme facility to throw away the product from the catalytic site. In this context, previous studies emphasized the importance of the active site flexibility for substrate recognition, catalysis and product release (Sanjeev and Vishveshwara, 2005; Gagné et al., 2012; Gagné and Doucet, 2013). The authors identified two main clusters involved in the protein motion that participate in substrate recognition and product release. In particular, L4 loop plays a key role in the protein motion (Gagné et al., 2012). In addition, a distant residue, Ala109, was identified in RNase A to work as a hinge and promote the active cleft opening and product release (Gagné et al., 2015). Ala109 is conserved in almost all the studied vertebrate members, except in frog and chicken RNases. To note, chicken family members are characterized by a much lower catalytic efficiency. On the other hand, comparison of zebrafish proteins indicates that presence of an Ala or Gly at this position is associated to high catalytic efficiency (Kazakou et al., 2008). On the other hand, a network of sequential hydrogen bond interactions was found mostly dependent on His48 protonation state, where deprotonation is associated to product release (Doucet et al., 2009; Watt et al., 2011). Interestingly, His48 is close to the protein family signature CKXXNTF and is conserved in most members, except in fish and amphibian sequences (**Figures S7**–**S11**).

Early dynamic predictions could also clearly differentiate between the main B2 residue (Asn71), which directly interacted with the adenine base, and other contributing residues, such as residues Gln69 and Glu111, which participated through water-mediated interactions (Seshadri et al., 1995; Madhusudhan and Vishveshwara, 2001). The results helped to interpret previous results obtained by site-directed mutagenesis and kinetic characterization (Tarragona-Fiol et al., 1993). Likewise, the NMR analysis of several frog RNases in complex with a deoxytetranucleotide also highlighted the key role of Asn71 counterparts for guanine binding, even if the respective L4 loops are significantly shortened in contrast to the bovine RNase A structure (Hsu et al., 2015). Moreover, the studies by Hsu and Chen corroborated the importance of Glu111 counterpart in specific guanine recognition at the N1/N2 group (Hsu et al., 2015).

On the other hand, significant divergence is evidenced at the guanine-binding mode between the present molecular dynamics analysis and previous structural characterization by X-ray crystallography. Mostly, although our data emphasizes the preference for adenine at B2 site in mammal RNases, we do not observe any impediment for guanine positioning at the enzyme base secondary site, nor any tendency of guanine to bind at the main B1 base site. Surprisingly, RNase A crystallographic studies using 2′5′-UpG and d(UpG), both in soaking and co-crystallization conditions, showed an unusual binding mode (Lisgarten et al., 1995). Specifically, the guanine was located at B1 instead of B2 site; this peculiar non-productive positioning was classified as a “retrobinding” mode (Aguilar et al., 1992). In addition, not only was “retrobinding” reported by independent researchers for RNase A for both d(CpG) and d(UpG) (Aguilar et al., 1992; Lisgarten et al., 1995; Vitagliano et al., 2000), but also for bullfrog RNase binding to d(CpA) (Chang et al., 1998). Noteworthy, the present kinetic results are also emphasizing a much more pronounced substrate selectivity at B2 site than the MD data reveal (**Table 1**, **Figure 2**).

**Figure 6:**
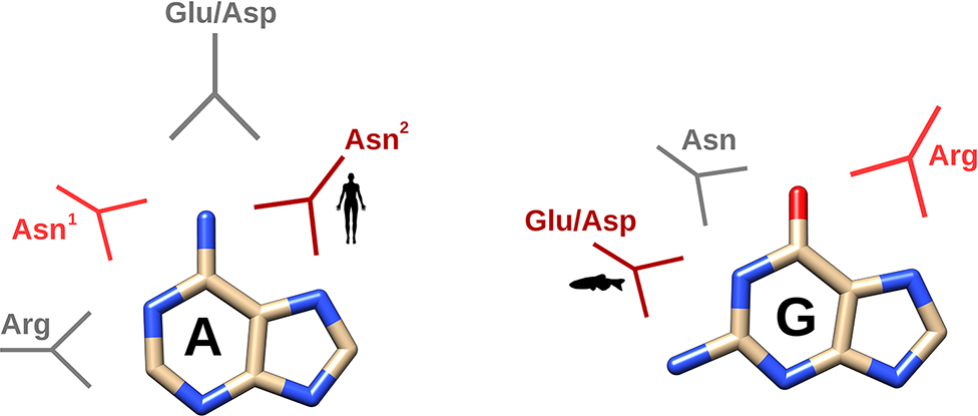
Schematic depiction of the main interactions identified by molecular dynamics according to the two main family classes (mammalian versus non-mammalian RNases). In dark and light red, the main and secondary specific interactions characteristic for either mammalian or non-mammalian RNases and in grey, the other interactions observed in all the RNases analyzed. Representative icon labels for humans and fishes illustrate the predominant interaction mode for each RNase class. Asn^1^ corresponds to Asn71 and Asn^2^ to Asn 67 in RNase A.

Overall, our molecular dynamics study using UpA and UpG enabled us to outline the main residues involved in the RNases’ distinct specificities for B2. **Figure 5** illustrates the main interactions that participate in the purine recognition. First, bidentate interactions can mainly discriminate between binding to either adenine or guanine at N1/N6 or N1/O6 groups respectively. In addition, we observe specific interactions at N7/N6 for adenine versus N7/O6 for guanine; and eventually specific binding at guanine N1/N2 group. A summary of the most representative residues that provide selectivity for each base is shown in **Figure 6**. Although no universal rules can be written for protein-nucleotide base binding, the residues identified in our study for RNase A superfamily members match most of the previously reported in the literature (Luscombe, 2001; Kondo and Westhof, 2011). Our previous statistical analysis of protein–nucleotide complexes available at the Protein Data Bank also highlighted the main contribution of Asn/Gln, Arg and Glu/Asp that provide bidentate interactions at N1/N6 and N1/O6 or N1/N2 groups, respectively (Boix et al., 2013). Other polar or charged secondary residues, such as Thr, Ser or Lys could also be identified (Luscombe, 2001; Boix et al., 2013). Complementarily, stacking interactions are also significantly influencing the protein binding mode (Luscombe, 2001; Boix et al., 2013). Interestingly, another structural feature reported by Westhof and co-workers as characteristic for adenine binding is the combined contribution of amino acid side chain and the peptide backbone atoms (Kondo and Westhof, 2011). Our molecular dynamics analysis highlights the conserved binding mode for adenine of Asn71 in RNase A, and counterparts, together with L4 loop main chain atoms. This emphasizes the importance of Asn and loop L4 conformation in RNase A superfamily to favor adenine binding in mammals (**Figure 4**). On their turn, lower order vertebrates tend to present an Arg that facilitates the interactions at N7/O6 for guanine recognition, as reported for other nucleotide-binding proteins (Luscombe, 2001).

Last, together with the two natural purine bases found in RNA we decided here to analyse the modified base inosine. Inosine molecular structure was used as a purine binding model that served to visualize unique interactions at N7/O6 and N1/O6, in relation to guanine. Comparison of kinetic and MD results on UpG and UpI highlights the importance of specific Glu/Asp residues in non-mammalian RNases involved in guanine N1/N2 group recognition. Interestingly, we find in the literature an inosine-specific RNase that can accommodate the base in its active site groove and provides specificity by discriminating the modified base against the two natural purines (Versées et al., 2002). To note, the contribution of Trp side chain in packing the inosine base by stacking interactions is observed. Inosine represents one of the main posttranscriptional modifications in cellular transcripts. RNA modifications not only contribute to regulate the translation pathway, they are also involved in the generation of regulatory tRNA fragments (Lyons et al., 2018). It is important to highlight that specific tRNA cleavage participates in the host response in stress conditions (Thompson et al., 2008) and RNA posttranscriptional modification can alter the target specificity for cellular endonucleases. For example, base methylation can protect tRNA from cleavage by human RNase 5 (angiogenin) (Lyons et al., 2017). Overall, RNA modifications not only alter their own processing rate but also influence their association to selective binding proteins, participating in the cellular metabolism and physiology (Boccaletto et al., 2017). Besides, the complexity of cellular RNA structure and its organization into supramolecular complexes within the cell further difficult our understanding of the cellular RNA metabolism (Van Treeck et al., 2018). Definitely, we are still facing important methodological limitations to interpret the RNases’ behavior in physiological conditions.

On the other hand, a comprehensive analysis of the protein nucleotide recognition pattern cannot disregard the existence of an extended substrate binding site architecture as demonstrated by many structural and kinetic studies (Boix et al., 1994; Fontecilla-Camps et al., 1994; Irie et al., 1998; Nogués et al., 1998; Raines, 1998; Hsu et al., 2015; Prats-Ejarque et al., 2019). Interestingly, recent work on the protein motion and ligand binding energies using a pentanucleotide suggests that induced conformational changes take place upon RNA interaction with secondary binding sites and can eventually provide a synergistic addition effect (Narayanan et al., 2017). The cooperative participation of secondary substrate binding sites could explain the enzyme low binding affinity for mono- and dinucleotides and is also significantly limiting the potency of molecular dynamics predictions, when working with such probes. However, our present results, together with previously reported data, are definitely indicating an evolutionary trend in B2 base selectivity within the vertebrate-specific RNase A superfamily that should respond to changing environmental conditions and adaptation to novel physiological needs. There is still a long path to walk to unveil the RNases’ substrate selectivity *in vivo*. We are confident that the identification of the structural patterns for nucleotide recognition in host defense RNases would provide valuable tools for structure-based drug design.

## Conclusions

In this work, we have analysed the base selectivity at B2 site within the RNase A superfamily by kinetic assays and molecular dynamics simulations using dinucleotide substrates. Our results indicate an evolutionary drift tendency from guanine to adenine preference. Interestingly, a close inspection of the residues potentially involved in the enzyme B2 site reveals that the main contributors (Asn71 and Glu111 in RNase A and equivalent counterparts) are present in all the family members. Notwithstanding, significant differences in L4 loop extension and contribution of complementary residues can facilitate a distinct binding mode that confers discrimination between both purine bases. Overall, Asn, Glu/Asp and Arg bidentate side chains provide selective binding to adenine N1/N6 and N6/N7 versus guanine N1/O6, O6/N7 and N1/N2 groups.

## Data Availability Statement

All datasets generated for this study are included in the manuscript/**Supplementary Files**.

## Author Contributions

EB and GP-E conceived and designed the experiments. GP-E, LL, VS and MM performed the experimental work. EB, GP-E and LL analysed the data. EB and GP-E drafted the manuscript. EB, GP-E, LL, VS and MM revised the final manuscript. All authors approved the final manuscript version.

## Funding

Research work was supported by the Ministerio de Economía y Competitividad (SAF2015-66007P) and by AGAUR, Generalitat de Catalunya (2016PROD00060; 2017SGR1010), co-financed by FEDER funds and by Fundació La Marató de TV3 (20180310). GP-E is a recipient of a PIF (UAB) predoctoral fellowship. LL is a recipient of a CSC predoctoral fellowship.

## Conflict of Interest

The authors declare that the research was conducted in the absence of any commercial or financial relationships that could be construed as a potential conflict of interest.

## Supplementary Material

Supplementary Figure 1 Modifications of the force field to include inosine parametrization.

Supplementary Figure 2 Phylogenetic tree of representative sequences of pancreatic ribonucleases. The evolutionary history was inferred by using the Maximum Likelihood method and JTT matrix-based model (Jones et al., 1992). The tree with the highest log likelihood (−27534.43) is shown. The percentage of trees in which the associated taxa clustered together is shown next to the branches. Initial tree(s) for the heuristic search were obtained automatically by applying Neighbour-Join and BioNJ algorithms to a matrix of pairwise distances estimated using a JTT model, and then selecting the topology with superior log likelihood value. The tree is drawn to scale, with branch lengths measured in the number of substitutions per site. This analysis involved 160 amino acid sequences. There were a total of 212 positions in the final dataset. Evolutionary analyses were conducted in MEGA X (Kumar et al., 2018). RNases are labeled with the species abbreviation (see **Table S1**) and its UNIPROT code, or, in its absence, with its NCBI code.

Supplementary Figure 3 Atom nomenclature of the three dinucleotides used in the molecular dynamics simulations.

Supplementary Figure 4 Mobility of the dinucleotides, calculated in RMSD (nm), during each 100 ns simulation run. Each color represents a different replicate.

Supplementary Figure 5 Fraction of hydrogen bond interaction occurrence of the key protein residues involved in the binding to the purine base during each MD simulation run.

Supplementary Figure 6 Schematic illustration of RNase-UpG complexes obtained by molecular dynamics simulations using GROMACS. The picture was generated using PyMOL 1.7.2 (Schrödinger, Inc).

Supplementary Figure 7 Sequence alignment of representative sequences of fish RNases. Protein regions identified to participate in B2 site are highlighted in yellow (L4, spanning from b2 to b3, end of ß6 and one of the two catalytic histidines together with a close by residue at ß7). Main conserved key residues are: Asn 72/74, Glu 114 and Glu 122/Arg123. TT indicates the presence of a ß-turn. Dots label every 10 residues of the reference protein used (Dr-RNase 1). The disulphide bonds are labeled with green numbers. The alignment was performed using Clustal Omega (Sievers and Higgins, 2018), and the picture was drawn using ESPript (Robert and Gouet, 2014). Labels are as follows: red box, white character for strict identity; red character for similarity within a group; and character with blue frame for similarity across groups.

Supplementary Figure 8 Sequence alignment of representative sequences of amphibian RNases. Protein regions identified to participate in B2 site are highlighted in yellow (L4, spanning from b2 to b3, end of ß6 and one of the two catalytic histidines together with a close by residue at ß7). Main conserved key residues are: Arg5, Asn56 and Thr89/Glu91. TT indicates the presence of a ß-turn. Dots label every 10 residues of the reference protein used (Rp-RNase). The disulphide bonds are labelled with green numbers. The alignment was performed using Clustal Omega (Sievers and Higgins, 2018), and the picture was drawn using ESPript (Robert and Gouet, 2014). Labels are as follows: red box, white character for strict identity; red character for similarity within a group; and character with blue frame for similarity across groups.

Supplementary Figure 9 Sequence alignment of representative sequences of reptilian RNases. Protein regions identified to participate in B2 site are highlighted in yellow (L4, spanning from b2 to b3, end of ß6 and one of the two catalytic histidines together with a close by residue at ß7). Main conserved key residues are: Asn68 and Asp116/Arg117. TT indicates the presence of a ß-turn. Dots label every 10 residues of the reference protein used (Cm-RNase 1). The disulphide bonds are labelled with green numbers. The alignment was performed using Clustal Omega (Sievers and Higgins, 2018), and the picture was drawn using ESPript (Robert and Gouet, 2014). Labels are as follows: red box, white character for strict identity; red character for similarity within a group; and character with blue frame for similarity across groups.

Supplementary Figure 10 Sequence alignment of representative sequences of bird RNases. Protein regions identified to participate in B2 site are highlighted in yellow (L4, spanning from b2 to b3, end of ß6 and one of the two catalytic histidines together with a close by residue at ß7). Main conserved key residues are: Asn65, Arg66 and Trp105. TT indicates the presence of a ß-turn. Dots label every 10 residues of the reference protein used (Gg-RNase 1). The disulphide bonds are labelled with green numbers. The alignment was performed using Clustal Omega (Sievers and Higgins, 2018), and the picture was drawn using ESPript (Robert and Gouet, 2014). Labels are as follows: red box, white character for strict identity; red character for similarity within a group; and character with blue frame for similarity across groups.

Supplementary Figure 11 Sequence alignment of representative sequences of mammalian RNases. Protein regions identified to participate in B2 site are highlighted in yellow (L4, spanning from b2 to b3, end of ß6 and one of the two catalytic histidines together with a close by residue at ß7). Main conserved key residues are: Asn67/Gln69/Asn71, Ala109, Glu111 and Arg122. TT indicates the presence of a ß-turn. Dots label every 10 residues of the reference protein used (Bt-RNase 1). The disulphide bonds are labelled with green numbers. The alignment was performed using Clustal Omega (Sievers and Higgins, 2018), and the picture was drawn using ESPript (Robert and Gouet, 2014). Labels are as follows: red box, white character for strict identity; red character for similarity within a group; and character with blue frame for similarity across groups.

Supplementary Table 1 Phylogenetic classification and abbreviations used for the analysed species.

Supplementary Table 2 Main key residues identified by molecular dynamic simulations of the studied RNase complexes with UpA, UpG and UpI. Equivalent residues by structural superposition are indicated. Cationic residues are indicated in blue, anionic residues in red and polar residues in black. Histidine residues involved in stacking interactions with the purine base are coloured in orange. Only specific interactions with purine atoms are included. Specific interaction to purine atoms are illustrated in **Figure 5**.
