# Supplementary figures and images for: Evolutionary Trends in RNA Base Selectivity Within the RNase A Superfamily
Source: Front Pharmacol. 2019 Oct 9;10:1170. doi: 10.3389/fphar.2019.01170 (PMC6794472; doi:10.3389/fphar.2019.01170)

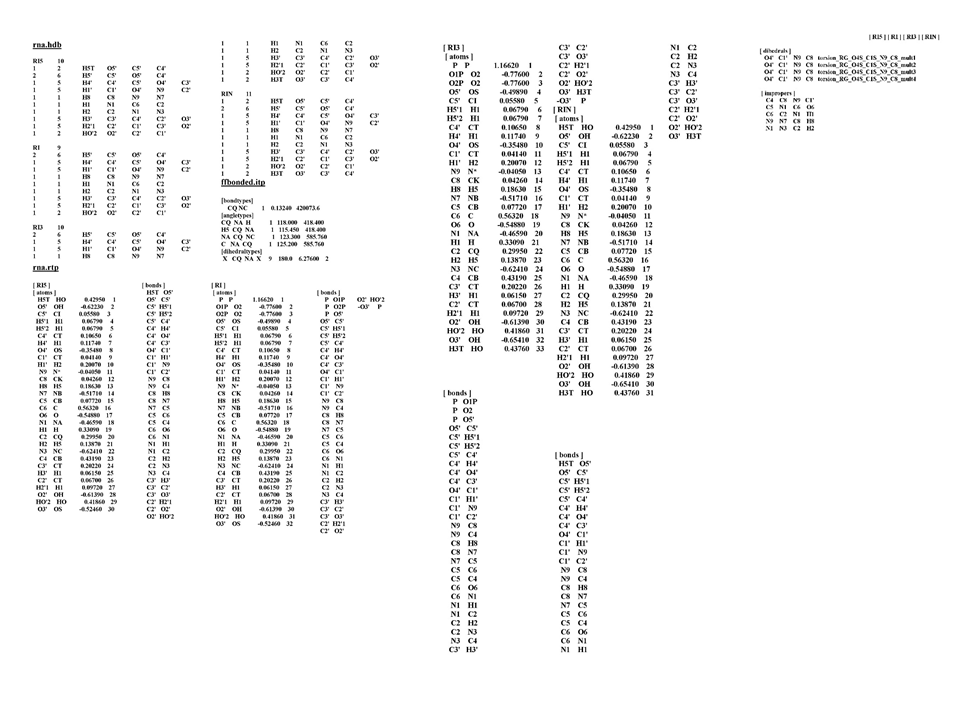

Supplement: Supplementary Figure 1 — Modifications of the force field to include inosine parametrization. [file Image_1.tif]

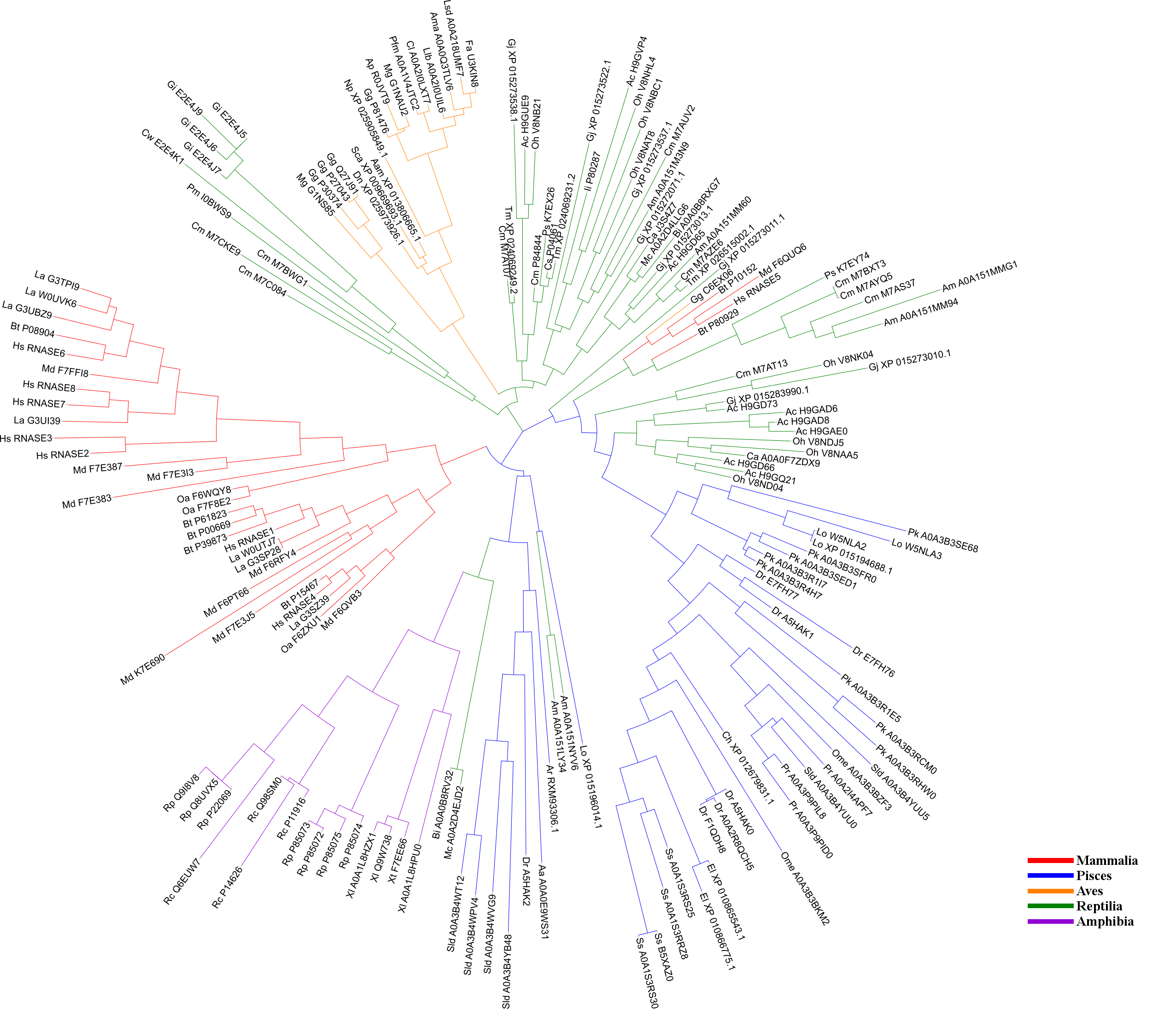

Supplement: Supplementary Figure 2 — Phylogenetic tree of representative sequences of pancreatic ribonucleases. The evolutionary history was inferred by using the Maximum Likelihood method and JTT matrix-based model (Jones et al., 1992). The tree with the highest log likelihood (−27534.43) is shown. The percentage of trees in which the associated taxa clustered together is shown next to the branches. Initial tree(s) for the heuristic search were obtained automatically by applying Neighbour-Join and BioNJ algorithms to a matrix of pairwise distances estimated using a JTT model, and then selecting the topology with superior log likelihood value. The tree is drawn to scale, with branch lengths measured in the number of substitutions per site. This analysis involved 160 amino acid sequences. There were a total of 212 positions in the final dataset. Evolutionary analyses were conducted in MEGA X (Kumar et al., 2018). RNases are labeled with the species abbreviation (see Table S1 ) and its UNIPROT code, or, in its absence, with its NCBI code. [file Image_2.jpeg]

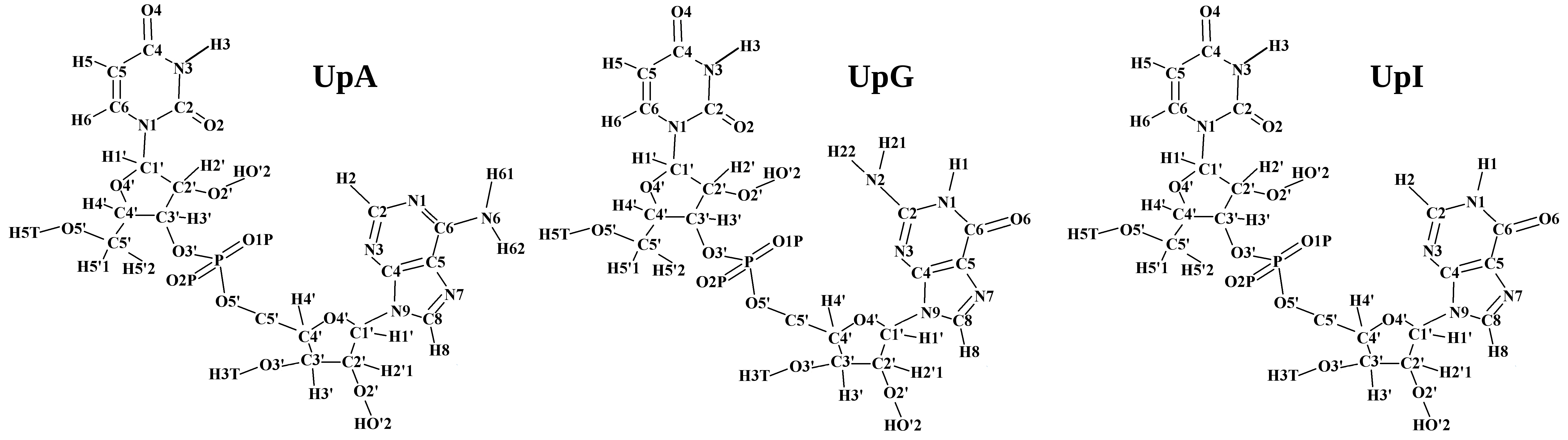

Supplement: Supplementary Figure 3 — Atom nomenclature of the three dinucleotides used in the molecular dynamics simulations. [file Image_3.jpeg]

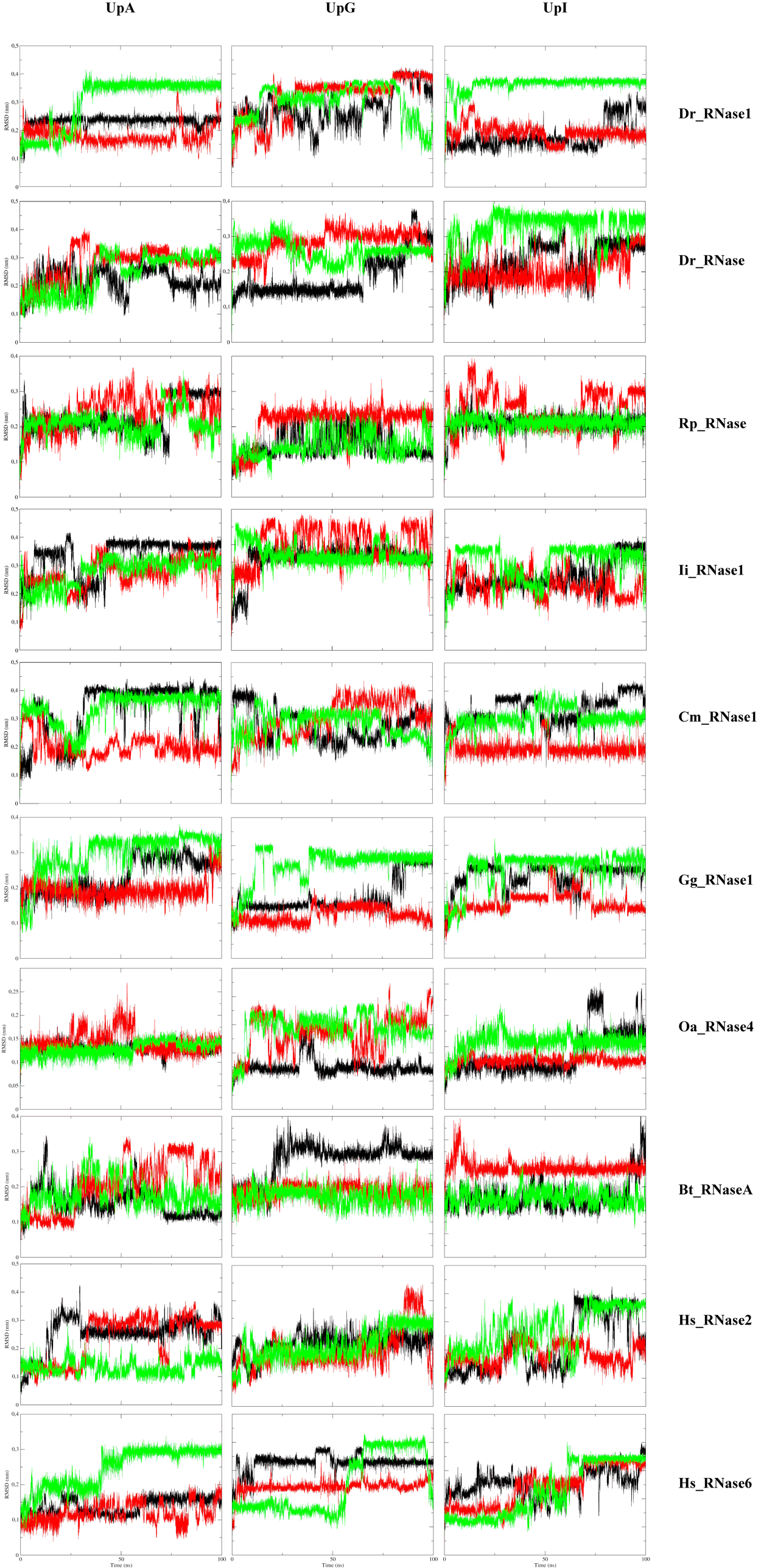

Supplement: Supplementary Figure 4 — Mobility of the dinucleotides, calculated in RMSD (nm), during each 100 ns simulation run. Each color represents a different replicate. [file Image_4.jpeg]

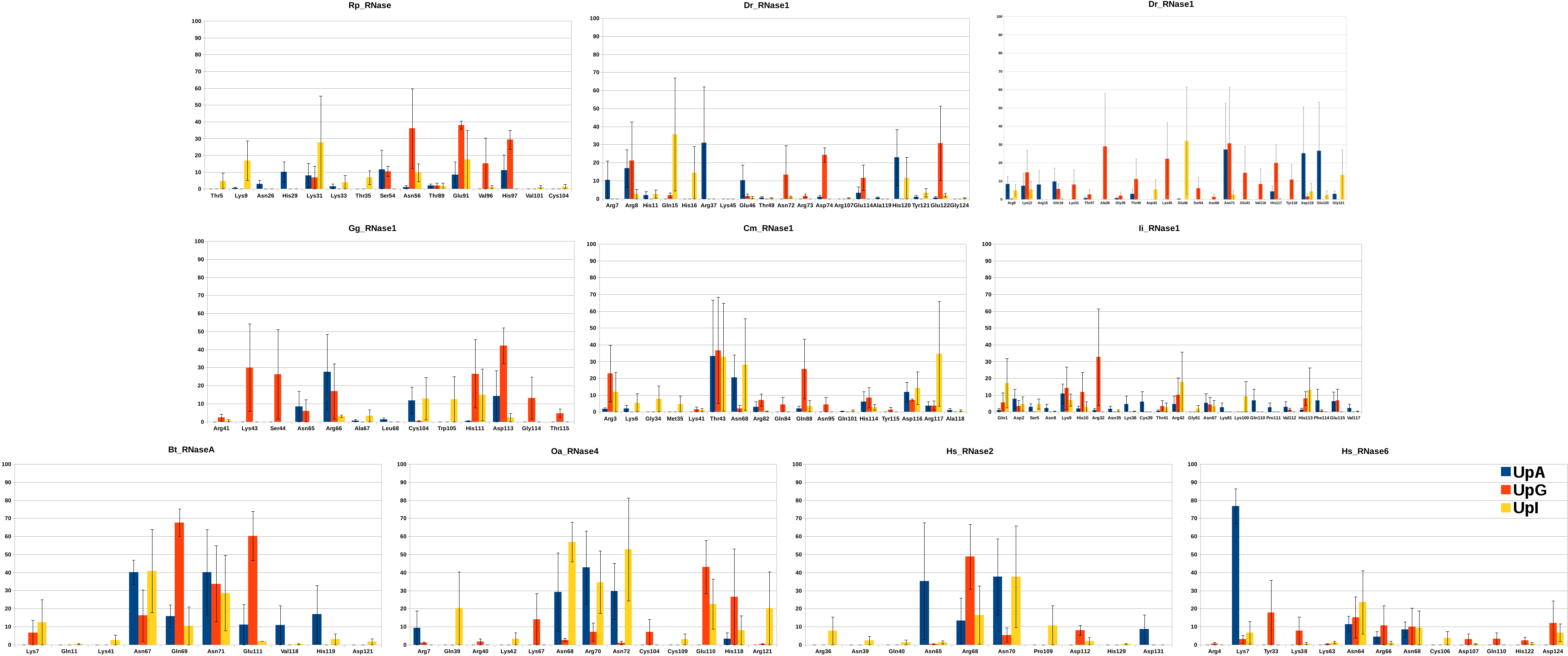

Supplement: Supplementary Figure 5 — Fraction of hydrogen bond interaction occurrence of the key protein residues involved in the binding to the purine base during each MD simulation run. [file Image_5.jpeg]

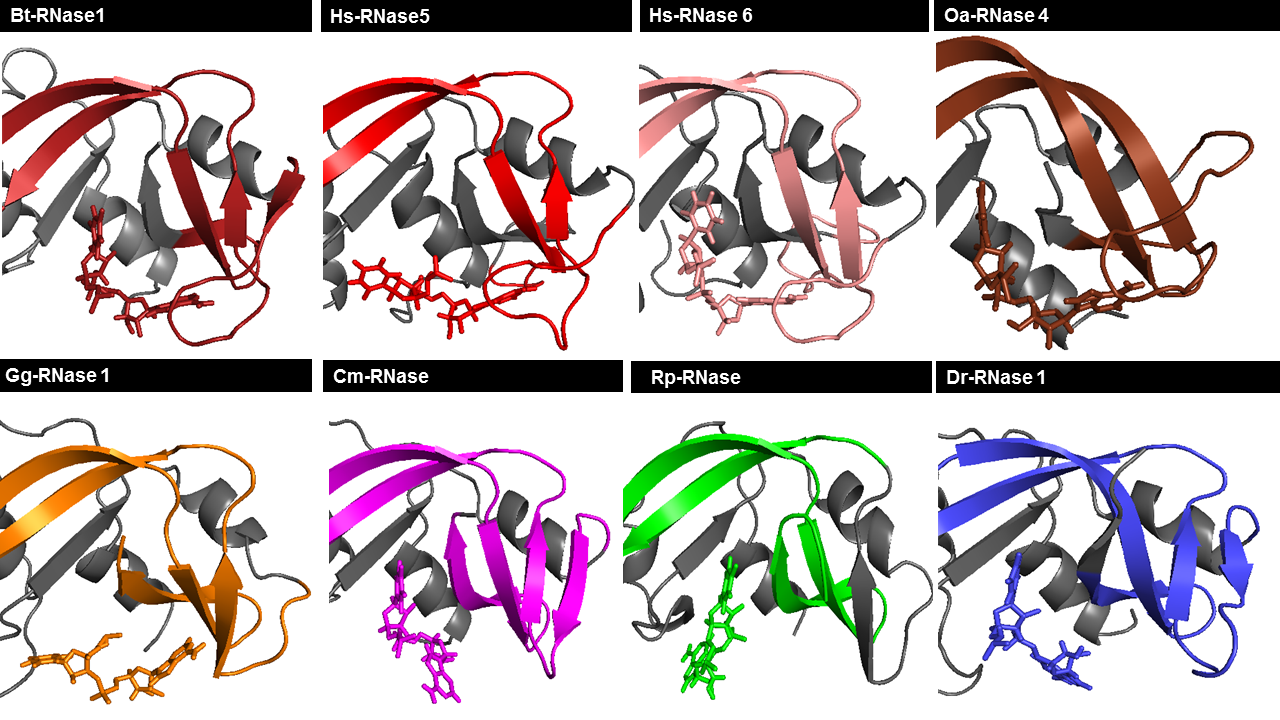

Supplement: Supplementary Figure 6 — Schematic illustration of RNase-UpG complexes obtained by molecular dynamics simulations using GROMACS. The picture was generated using PyMOL 1.7.2 (Schrödinger, Inc). [file Image_6.tif]

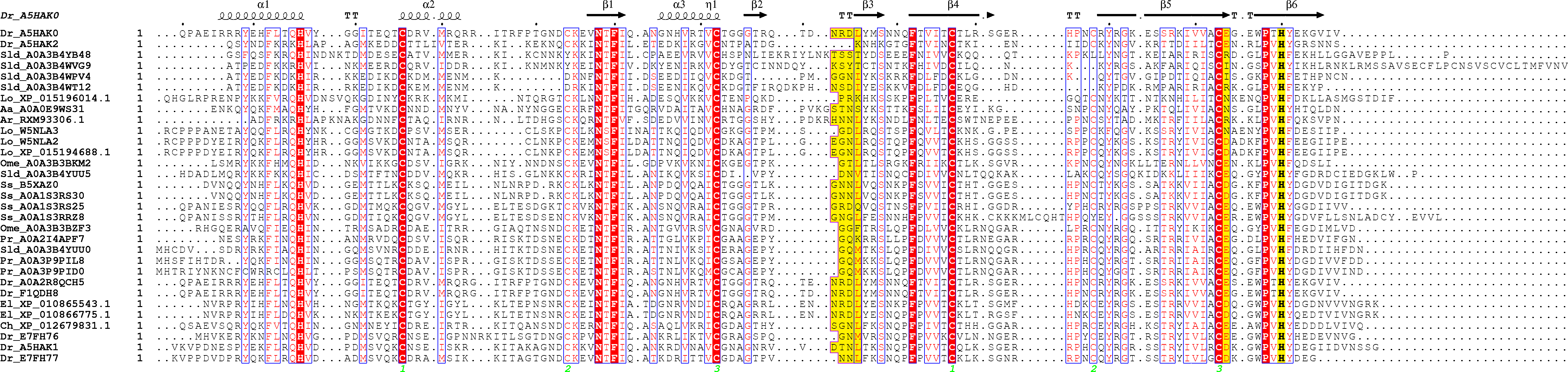

Supplement: Supplementary Figure 7 — Sequence alignment of representative sequences of fish RNases. Protein regions identified to participate in B2 site are highlighted in yellow (L4, spanning from b2 to b3, end of ß6 and one of the two catalytic histidines together with a close by residue at ß7). Main conserved key residues are: Asn 72/74, Glu 114 and Glu 122/Arg123. TT indicates the presence of a ß-turn. Dots label every 10 residues of the reference protein used (Dr-RNase 1). The disulphide bonds are labeled with green numbers. The alignment was performed using Clustal Omega (Sievers and Higgins, 2018), and the picture was drawn using ESPript (Robert and Gouet, 2014). Labels are as follows: red box, white character for strict identity; red character for similarity within a group; and character with blue frame for similarity across groups. [file Image_7.jpeg]

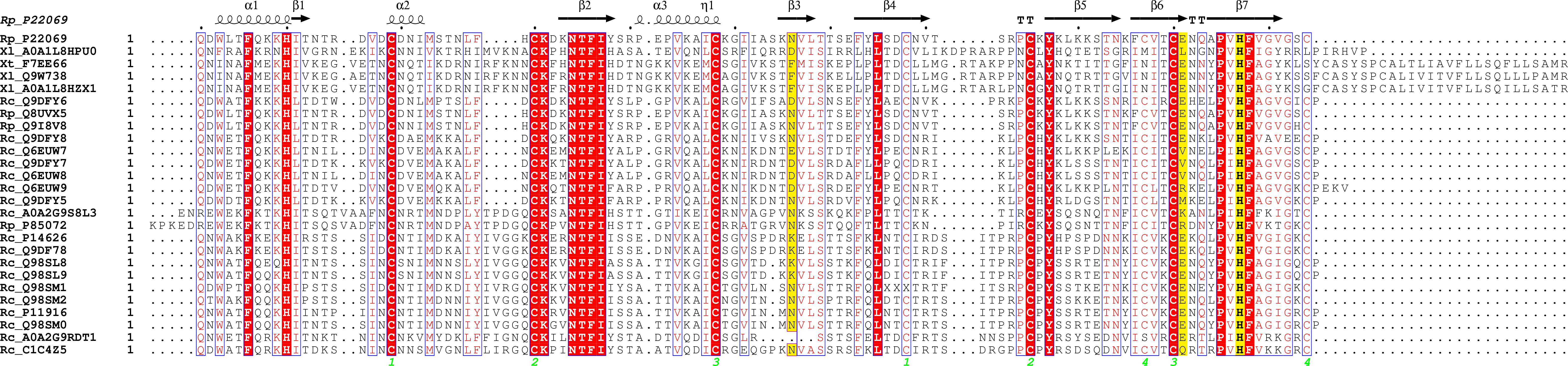

Supplement: Supplementary Figure 8 — Sequence alignment of representative sequences of amphibian RNases. Protein regions identified to participate in B2 site are highlighted in yellow (L4, spanning from b2 to b3, end of ß6 and one of the two catalytic histidines together with a close by residue at ß7). Main conserved key residues are: Arg5, Asn56 and Thr89/Glu91. TT indicates the presence of a ß-turn. Dots label every 10 residues of the reference protein used (Rp-RNase). The disulphide bonds are labelled with green numbers. The alignment was performed using Clustal Omega (Sievers and Higgins, 2018), and the picture was drawn using ESPript (Robert and Gouet, 2014). Labels are as follows: red box, white character for strict identity; red character for similarity within a group; and character with blue frame for similarity across groups. [file Image_8.jpeg]

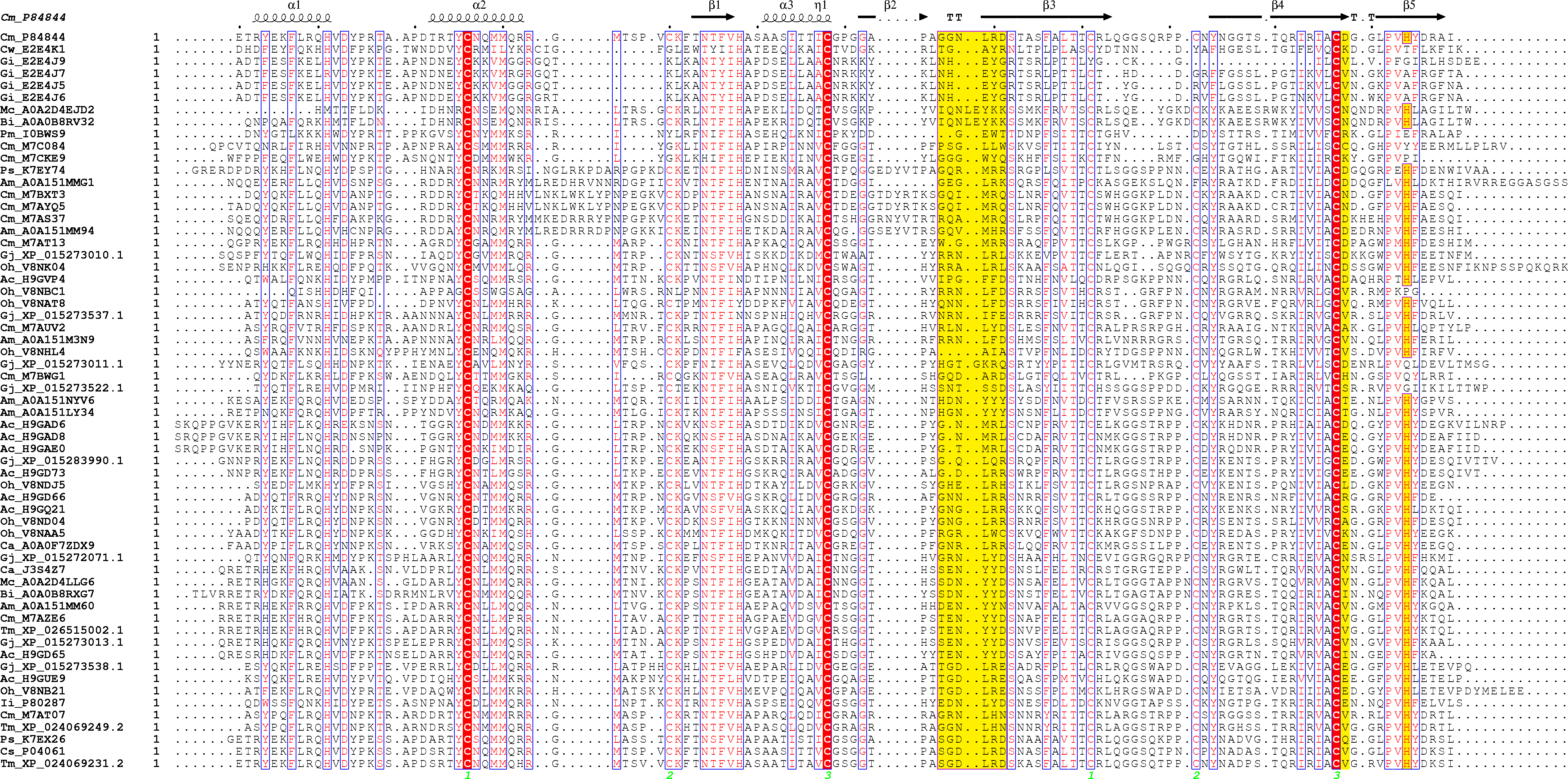

Supplement: Supplementary Figure 9 — Sequence alignment of representative sequences of reptilian RNases. Protein regions identified to participate in B2 site are highlighted in yellow (L4, spanning from b2 to b3, end of ß6 and one of the two catalytic histidines together with a close by residue at ß7). Main conserved key residues are: Asn68 and Asp116/Arg117. TT indicates the presence of a ß-turn. Dots label every 10 residues of the reference protein used (Cm-RNase 1). The disulphide bonds are labelled with green numbers. The alignment was performed using Clustal Omega (Sievers and Higgins, 2018), and the picture was drawn using ESPript (Robert and Gouet, 2014). Labels are as follows: red box, white character for strict identity; red character for similarity within a group; and character with blue frame for similarity across groups. [file Image_9.jpeg]

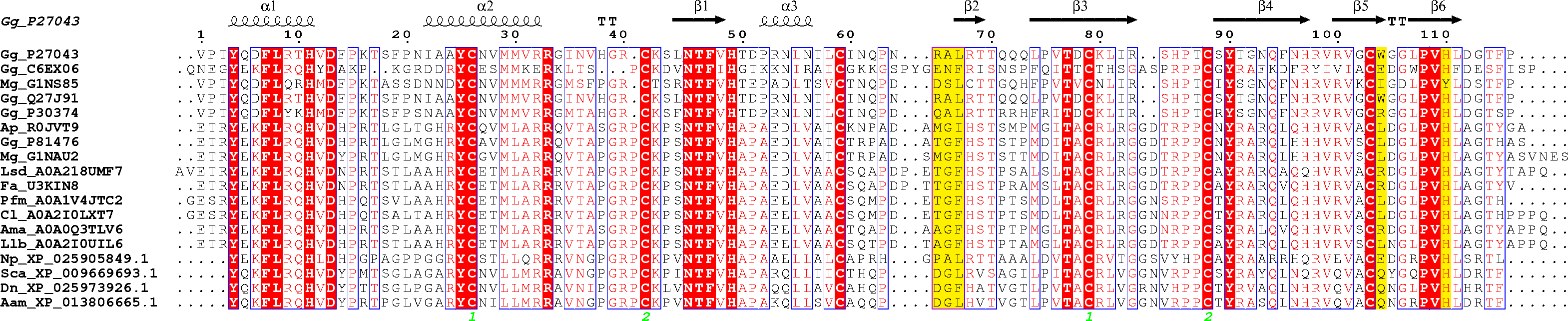

Supplement: Supplementary Figure 10 — Sequence alignment of representative sequences of bird RNases. Protein regions identified to participate in B2 site are highlighted in yellow (L4, spanning from b2 to b3, end of ß6 and one of the two catalytic histidines together with a close by residue at ß7). Main conserved key residues are: Asn65, Arg66 and Trp105. TT indicates the presence of a ß-turn. Dots label every 10 residues of the reference protein used (Gg-RNase 1). The disulphide bonds are labelled with green numbers. The alignment was performed using Clustal Omega (Sievers and Higgins, 2018), and the picture was drawn using ESPript (Robert and Gouet, 2014). Labels are as follows: red box, white character for strict identity; red character for similarity within a group; and character with blue frame for similarity across groups. [file Image_10.jpeg]

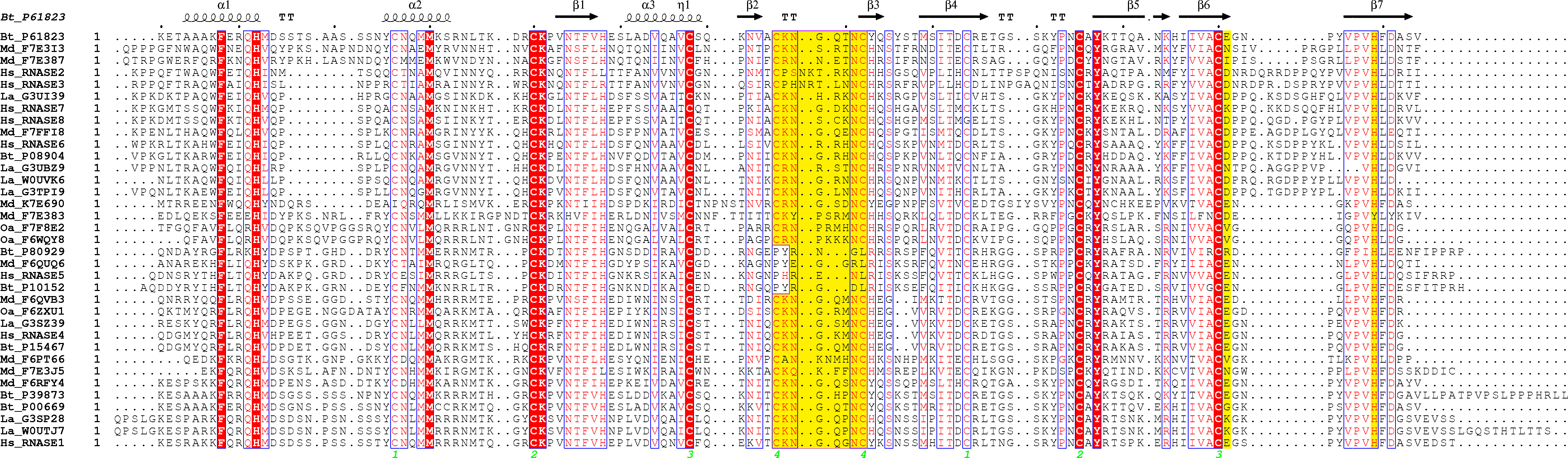

Supplement: Supplementary Figure 11 — Sequence alignment of representative sequences of mammalian RNases. Protein regions identified to participate in B2 site are highlighted in yellow (L4, spanning from b2 to b3, end of ß6 and one of the two catalytic histidines together with a close by residue at ß7). Main conserved key residues are: Asn67/Gln69/Asn71, Ala109, Glu111 and Arg122. TT indicates the presence of a ß-turn. Dots label every 10 residues of the reference protein used (Bt-RNase 1). The disulphide bonds are labelled with green numbers. The alignment was performed using Clustal Omega (Sievers and Higgins, 2018), and the picture was drawn using ESPript (Robert and Gouet, 2014). Labels are as follows: red box, white character for strict identity; red character for similarity within a group; and character with blue frame for similarity across groups. [file Image_11.jpeg]
