# Supplementary material for: Evolutionary Trends in RNA Base Selectivity Within the RNase A Superfamily
Source: Front Pharmacol. 2019 Oct 9;10:1170. doi: 10.3389/fphar.2019.01170 (PMC6794472; doi:10.3389/fphar.2019.01170)
Supplement: Supplementary Table 1 — Phylogenetic classification and abbreviations used for the analysed species. [file Table_1.docx]

| **Class** | **Subclass** | **Cohort** | **Subcohort** | **Order** | **Suborder/**  **Infraorder** | **Family** | **Species** | **Abb.** |
| --- | --- | --- | --- | --- | --- | --- | --- | --- |
| Actinopteri  (Pisces) | Neopterygii | Elopomorpha | - | Anguilliformes | - | Anguillidae | *Anguilla anguilla* | Aa |
|  |  | Otomorpha | Ostariophysi | Cypriniformes | Cyprinoidei | Cyprinidae | *Danio rerio* | Dr |
|  |  |  | Clupei | Clupeiformes | Clupeoidei | Clupeidae | *Clupea harengus* | Ch |
|  |  | Euteleostei | Protacanthopterygii | Salmoniformes | - | Salmonidae | *Salmo salar* | Ss |
|  |  |  |  | Esociformes | - | Esocidae | *Esox lucius* | El |
|  |  |  | Neoteleostei | Cyprinodontiformes | Cyprinodontoidei | Poeciliidae | *Poecilla reticulata* | Pr |
|  |  |  |  | Carangiformes | - | Carangidae | *Seriola lalandi dorsalis* | Sld |
|  |  |  |  | Beloniformes | Adrianichthyoidei | Adrianichthyidae | *Oryzias melastigma* | Ome |
|  |  | **-** | | Lepisosteiformes | - | Lepisosteidae | *Lepisosteus oculatus* | Lo |
|  | Chondrostei | - | | Acipenseriformes | - | Acipenseridae | *Acipenser ruthenus* | Ar |
| Amphibia | Lissamphibia | - | | Anura | Sokolanura | Ranidae | *Rana catesbeiana* | Rc |
|  |  |  |  |  |  |  | *Rana pipiens* | Rp |
|  |  |  |  |  | Xenoanura | Pipidae | *Xenopus laevis* | Xl |
| Reptilia | Diapsida | - | | Squamata | Gekkota | Gekkonidae | *Gekko japonicus* | Gj |
|  |  |  |  |  | Diploglossa | Diploglossidae | *Celestus warreni* | Cw |
|  |  |  |  |  |  | Anguidae | *Gerrhonotus infernalis* | Gi |
|  |  |  |  |  | Iguania | Iguanidae | *Iguana iguana* | Ii |
|  |  |  |  |  |  | Dactyloidae | *Anolis carolinensis* | Ac |
|  |  |  |  |  | Ophidia | Elapidae | *Ophiophagus hannah* | Oh |
|  |  |  |  |  |  |  | *Micrurus corallinus* | Mc |
|  |  |  |  |  |  | Lamprophiidae | *Psammophis mossambicus* | Pm |
|  |  |  |  |  |  | Viperidae | *Crotalus adamanteus* | Ca |
|  | Anapsida | - | | Testudines | Cryptodira | Cheloniidae | *Chelonia mydas* | Cm |
|  |  |  |  |  |  | Emydidae | *Terrapene mexicana* | Tm |
|  |  |  |  |  |  | Chelydridae | *Chelydra serpentina* | Cs |
|  |  |  |  | Crocodylia | Eusuchia | Alligatoridae | *Alligator mississippiensis* | Am |
|  | Aves | Neognathae | Galloanserae | Galliformes | - | Phasianidae | *Gallus gallus* | Gg |
|  |  |  |  |  |  |  | *Meleagris gallopavo* | Mg |
|  |  |  |  | Anseriformes | - | Anatidae | *Anas platyrhynchos* | Ap |
|  |  |  | Neoaves | Passeriformes | - | Muscicapidae | *Ficedula albicollis* | Fa |
|  |  |  |  |  |  | Estrildidae | *Lonchura striata domestica* | Lsd |
|  |  |  |  | Psittaciformes | - | Psittacidae | *Amazona aestiva* | Ama |
|  |  |  |  | Charadriiformes | - | Scolopacidae | *Limosa lapponica baueri* | Llb |
|  |  |  |  | Columbiformes | - | Columbidae | *Patagioenas fasciata monilis* | Pfm |
|  |  |  |  |  |  |  | *Columba livia* | Cl |
|  |  |  |  | Apterygiformes | - | Apterygidae | *Apteryx australis mantelli* | Aam |
|  |  | Paleognathae | - | Tinamiformes | - | Tinamidae | *Nothoprocta perdicaria* | Np |
| Mammalia | Prototheria | - | - | Monotremata | - | Ornithorhynchidae | *Ornithorhynchus anatinus* | Oa |
|  | Metatheria | - | - | Didelphimorphia | - | Didelphidae | *Monodelphis domestica* | Md |
|  | Theria | Placentalia | - | Proboscidea | Elephantiformes | Elephantidae | *Loxodonta africana* | La |
|  |  |  |  | Primates | Euprimates | Hominidae | *Homo sapiens* | Hs |
|  |  |  |  | Artiodactyla | Ruminantia | Bovidae | *Bos taurus* | Bt |

**Supplementary Table 1.** Phylogenetic classification and abbreviations used for the analysed species.
