# Supplementary material for: Evolutionary Trends in RNA Base Selectivity Within the RNase A Superfamily
Source: Front Pharmacol. 2019 Oct 9;10:1170. doi: 10.3389/fphar.2019.01170 (PMC6794472; doi:10.3389/fphar.2019.01170)
Supplement: Supplementary file 3 [file Table_2.docx]

RNaseA Reference numbering	Bt-RNaseA	Hs-RNase6	Hs-RNase2	Oa-RNase4	Cm-RNase	Ii-RNase	Gg-RNase	Rp-RNase	Dr-RNase
**A4**					R3			D2	R8
**R39**	R39								
**P42**						R42			E46
**V47**					T43				
**N67**	N67	N64	N65	N68					
**Q69**	Q69	R66	R68	R70				S54	N72
**N71**	N71	N68	N70	N72	N68	N67	R66	N56	D74
**G85**					Q88				
**E111**	E111	D107	D112	E110		E107		E91	E114
**H119**	H119	H122	H129	H118	H114	H113	H111	H97	H120
**D121**	D121	D124	D131		D116		D113		E122
**A122**			R132	R121	R117	E115			R123

Supplementary Table 2. Main key residues identified by molecular dynamic simulations of the studied RNase complex UpG and UpI. Equivalent residues by structural superposition are indicated. Cationic residues are indicated in blue, anion red and polar residues in black. Histidine residues involved in stacking interactions with the purine base are coloured in specific interactions with purine atoms are included. Specific interaction to purine atoms are illustrated in Figure 5.
